# Periosteum-inspired in situ CaP generated nanocomposite hydrogels with strong bone adhesion and superior stretchability for accelerated distraction osteogenesis

**DOI:** 10.1186/s40824-022-00330-1

**Published:** 2022-12-30

**Authors:** Tengfei Lou, Kai Chen, Qiyu Luo, Changsheng Liu, Yuan Yuan, Cunyi Fan

**Affiliations:** 1 Orthopaedic Department, Shanghai Sixth People’s Hospital, Shanghai, 200233 People’s Republic of China; 2 Key Laboratory for Ultrafine Materials of Ministry of Education, and School of Materials Science and Engineering, East China University of Science and Technology, Shanghai, 200237 People’s Republic of China; 3 Frontiers Science Center for Materiobiology and Dynamic Chemistry, and Engineering Research Center for Biomedical Materials of Ministry of Education, East China University of Science and Technology, Shanghai, 200237 People’s Republic of China

**Keywords:** In situ precipitated CaP nanoparticles, Nanocomposite hydrogels, Bone adhesion, Stretchability, Dynamic distraction osteogenesis, Mechanical stimulation

## Abstract

**Background:**

Distraction osteogenesis (DO) is an efficacious but lengthy procedure to reconstruct segmental bone defects under the principle of tension-stress, during which the periosteum-mediated mechanical stimulation plays a pivotal role. Inspired by the dynamic process of DO and the mechanical stimulation of periosteum, a new design of bionic periosteum was developed to simulate the mechanical transduction of natural periosteum for the application in DO procedure.

**Methods:**

In this study, an injectable organic-inorganic hybrid hydrogel was developed based on a novel combination of the PEGylated poly (glycerol sebacate) (PEGS) polymer network and in situ formed CaP nanoparticles (ICPNs). Rat bone marrow mesenchymal stem cells (rBMSCs) and human umbilical vein endothelial cells (HUVECs) were cultured and tested in vitro to evaluate biocompatibility, cell adhesion, proliferation, and pro-osteogenic and pro-angiogenic activity. In vivo experiments were conducted in the rat tibial model of distraction osteogenesis.

**Results:**

The developed nanocomposite hydrogels exhibited excellent injectability, robust bone adhesion, superior stretchability, and enhanced osteogenic activity. The results of in vitro and in vivo studies showed that PEGS/ICPN hydrogels could promote new bone formation and mineralization during the dynamic distraction process through the synergistic effects of angiogenesis and osteogenesis.

**Conclusions:**

This periosteum-inspired nanocomposite hydrogel represents a mechanobiology approach for effectively restoring large bone defects through the dynamic DO process.

**Supplementary Information:**

The online version contains supplementary material available at 10.1186/s40824-022-00330-1.

## Introduction

Distraction osteogenesis (DO) pioneered by Ilizarov is a biological technique for the restoration of large bone defects and has been comprehensively administered to the treatment of traumatic bone loss, osteomyelitis, non-union, bone tumors, and deformity [1–3]. The dynamic DO procedure mainly includes three phases that occur sequentially: the latency phase after osteotomy; the distraction phase in which the segmental ends are slowly and stably separated by continuous tension stress, creating a mechanobiological environment to stimulate new bone regeneration; and the consolidation phase for the maturation of the newly regenerated bone [1]. It is hypothesized that the slow dynamic process of DO resembles embryonic development, during which the preserved periosteum plays a pivotal role in transferring mechanical cues and further stimulating new bone formation in the distraction gap [2, 3]. However, coloboma of periosteum caused either by high-energy trauma or complicated surgical procedure commonly results in poor bone regeneration and further delays the treatment time of DO [4]. In recent years, investigators have made efforts to engineer bionic periosteum centered on biological factor or cell delivery to enhance bone regeneration [5]. However, these designs are commonly complicated by the concerns of biological safety, expensive cost, and sophisticated fabrication [6, 7]. In contrast, much less work has been done to imitate the mechanical stimulation transferred by the periosteum that can promote bone regeneration. And due to the unique dynamic process of DO, the application of biomedical materials with resembling functions to the natural periosteum remains research gap and great challenge.

Considering the dynamic feature of DO and the mechanical stimulation of periosteum, a bionic periosteum with strong bone adhesion and superior stretchability is expected to be an ideal biomedical material to substitute the nature periosteum and accelerate DO process. Injectable adhesive hydrogels have been extensively studied in biomedical field due to its similar physicochemical properties to natural extracellular matrix, desirable adhesion ability to biological tissues, and ease of operation in clinical practice [8–10]. Numerous studies have demonstrated that covalent bonding between the hydrogel network and tissue protein contributes to a robust adhesion [11, 12]. Based on this principle, various tissue adhesives with desirable moist and dynamic adhesion ability were developed, and some of them were applied as bone adhesives [13–16]. Meanwhile, several recent studies have demonstrated that in situ precipitation of calcium phosphate (CaP) in a hydrogel polymer network could simultaneously enhance the stretchability and osteogenic activity of the hydrogel due to the uniform CaP dispersion [17–19]. Therefore, it is hypothesized that an injectable, adhesive hydrogel with CaP nanoparticles formed in situ should be a more favorable artificial periosteum for accelerating DO process.

**Scheme 1:**
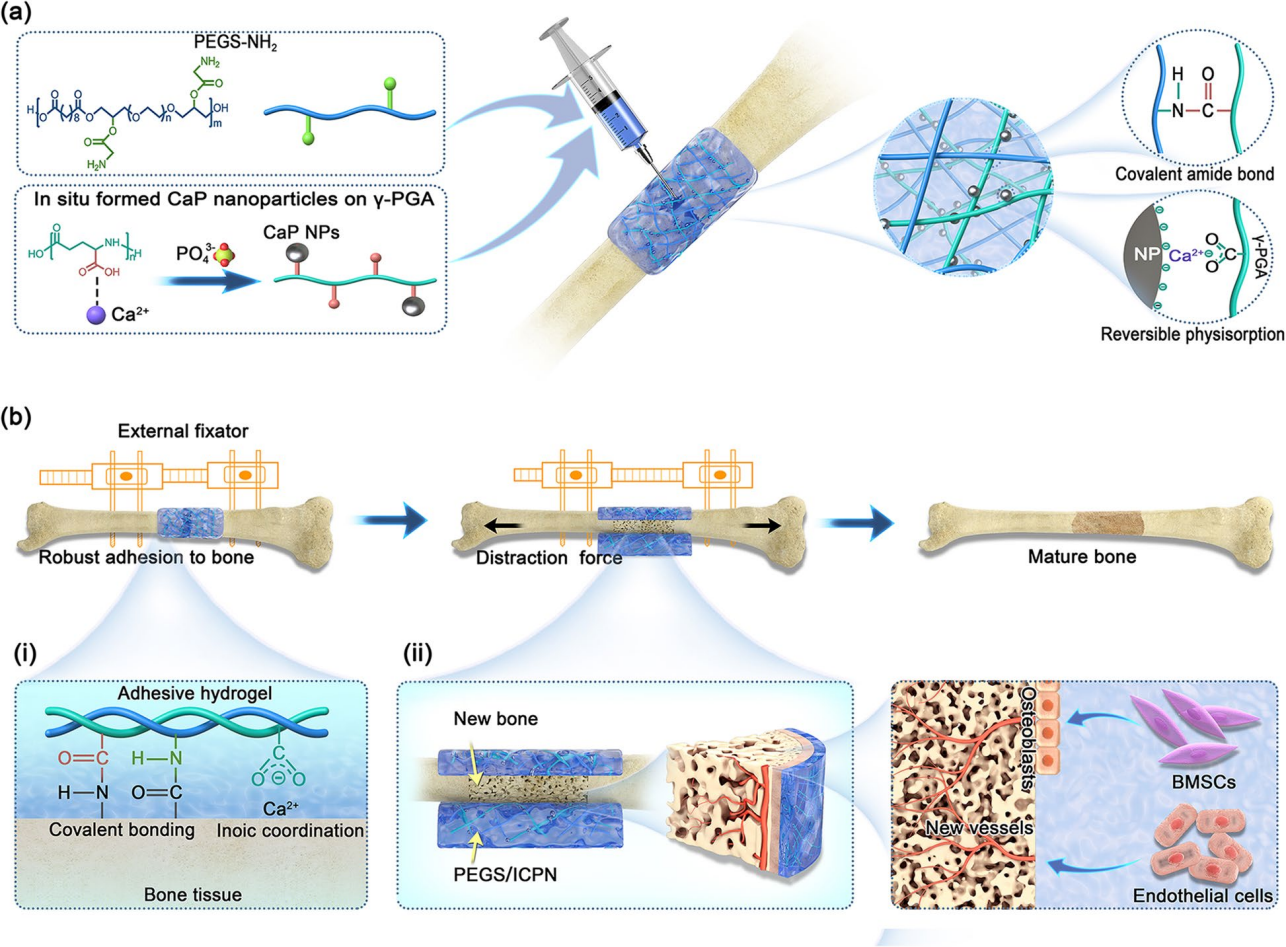
Schematic illustrations of design strategy for the PEGS/ICPN adhesive hydrogels and the application in distraction osteogenesis. a) Chemical structures of PEGS-NH2, γ-PGA and the process of in situ formation of CaP nanoparticles. PEGS-NH2 and γ-PGA can form chemically crosslinked structure in the presence of EDC/NHS via a coupling reaction. γ-PGA and the as-formed CaP nanoparticles can form physically crosslinked structure via γ-PGA chain-CaP nanoparticle physisorption together with -COO--Ca2+ ionic coordination. b) The process of distraction osteogenesis with the PEGS/ICPN hydrogels adhered to the zone of newly regenerative bone: (i) Multiple adhesion mechanisms formed between hydrogel and bone, including covalent amide bonds and -COO-- Ca2+ ionic coordination, contribute to the robust adhesion ability; (ii) The pro-osteogenic and pro-angiogenic effects of the PEGS/ICPN hydrogels in the DO process through recruitment and inducement of bone mesenchymal stem cells (BMSCs) and endothelial cells, enhancing regeneration and vascularization of the new bone

In this study, a periosteum-inspired injectable nanocomposite hydrogel with superior stretchability, strong bone adhesion ability, and enhanced osteogenic activity was developed based on a novel combination of the PEGylated poly (glycerol sebacate) (PEGS) polymer network and in situ formed CaP nanoparticles (ICPNs) for the dynamic DO procedure. PEGS, a biocompatible and biodegradable polyester with adequate functional hydroxyl groups, was modified with amino groups to form an organic matrix [16]. Poly(γ-glutamic acid) (γ-PGA) bearing abundant carboxyl groups was introduced as nucleation sites to induce the in situ precipitation of CaP nanoparticles via -COO^−^-Ca^2+^ ionic coordination. Meanwhile, a homogeneous and reversible physically crosslinked network was constructed based on γ-PGA chain-CaP nanoparticle physisorption. Then, the carboxyl groups on γ-PGA and the amino groups on PEGS can also form amide bonds to establish a chemically crosslinked network via a 1-ethyl-3-(3-dimethylaminopropyl)-carbodiimide hydrochloride/N-hydroxysuccinimide (EDC/NHS) coupling reaction. Relying on the double-crosslinked structures, the hydrogel is expected to exhibit superior stretchability (Scheme 1a). Moreover, the nanocomposite hydrogel can form covalent amide bonds and -COO^−^-Ca^2+^ ionic coordination with the carboxyl and amino groups and Ca^2+^ on the surface of bone, which collectively contribute to the robust adhesion to bone tissue (Scheme 1b(i)). Additionally, the obtained nanocomposite hydrogel further exhibited enhanced angiogenic and osteogenic activity in the dynamic DO procedure (Scheme 1b(ii)). This new adhesive, stretchable hydrogel is believed to serve as a promising artificial periosteum to promote mechanobiology-mediated bone regeneration.

## Materials and methods

### Materials

Sebacic acid (purity: 99%) and (NH_4_)_2_HPO_4_ (purity: 98.5%) were purchased from Shanghai Aladdin Biochemical technology Co., Ltd. Poly(ethylene glycol) diglycidyl ether (PEGDGE; average *M*_*n*_: 500 Da) was purchased from Sigma-Aldrich (Merck). Poly (γ-glutamic acid) (γ-PGA; average *M*_*n*_: ≈ 500 kDa) was purchased from Shanghai yuanye Bio-Technology Co., Ltd. Anhydrous solvents, including N, N-dimethylformamide (DMF; purity: 99.8%) and dichloromethane (DCM; purity: 99.9%), were purchased from Adamas-beta (Titan). Ca(NO_3_)_2_∙4H_2_O (purity: 98.5%) was purchased from Sinopharm Chemical Reagent Co., Ltd. All other chemical reagents were purchased from Shanghai Aladdin Biochemical Technology Co., Ltd. and used without further purification unless otherwise stated. BMSCs were derived from 2-week-old SD rat by rinsing the bone marrow cavity from bilateral femurs and tibias. HUVECs were obtained from Sciencell Research Laboratories (San Diego, CA, USA). Fetal bovine serum (FBS), α-minimum essential medium (α-MEM), trypsin–EDTA free (0.25%), penicillin–streptomycin (PS) were purchased from Gibco (Grand Island, NY, the USA). Endothelial cell medium (ECM) was purchased from ScienCell (Carlsbad, CA, USA). Calcein-AM/PI and CCK-8 were purchased from Biyuntian (Shanghai, China). TRIzol was purchased from Invitrogen (Carlsbad, CA, USA). All the antibodies were obtained from Abcam Biotechnology (Cambridge, MA). SD rats were supplied by Shanghai Sixth People’s Hospital (Shanghai, China).

### Preparation and characterization of PEGS/ICPN hydrogel

#### Synthesis of PEGylated poly (glycerol sebacate) (PEGS)

PEGS was synthesized based on an acid initiated epoxide ring-opening reaction. Before the synthesis of PEGS, we first prepared a catalyst, bis(tetrabutylammonium) sebacate (TBAS), as previously reported. Briefly, sebacic acid (0.98 g) and tetrabutylammonium hydroxide (TBAH; 10.00 g, ~ 25 wt% in H_2_O) were dissolved in 95% ethanol (50 ml) and reacted for 30 min at 55 °C under continuous stirring. The mixture was treated with rotary evaporation and lyophilization sequentially to obtain TBAS as a white powder. Then, sebacic acid (5.06 g), PEGDGE (12.50 g) and the catalyst TBAS (0.11) were dissolved in anhydrous DMF (50 ml) under nitrogen protection and stirred for 72 h at 100 °C. The mixture was precipitated in pre-cooled ethyl ether and dried overnight at 25 °C under vacuum to obtain PEGS.

#### Synthesis of amino-functionalized PEGS (PEGS-NH_2_)

PEGS-NH_2_ was synthesized as previously reported [16]. Briefly, PEGS (3.51 g), Boc-glycine (2.10 g), N, N′-diisopropylcarbodiimide (DIC; 2.53 g), and 4-dimethylaminopyridine (DMAP; 0.06 g) were dissolved in anhydrous DCM (50 ml) under nitrogen protection and stirred for 24 h at room temperature. The mixture was filtered and concentrated followed by treatment with trifluoroacetic acid (TFA; Macklin, Shanghai; 8 ml) for 30 min under vigorous stirring. Then, the crude product was dialyzed (MWCO: 3500; Viskase, USA) and dried under vacuum to obtain the purified PEGS-NH_2_.

#### Molecular structural characterizations

Proton nuclear magnetic resonance (^1^H NMR) spectroscopy (Bruker Ascend 600 MHz, Bruker Corporation, Switzerland) in dimethyl sulfoxide-d6 (DMSO-d6) was used to characterize the molecular structures of TBAS, PEGS and PEGS-NH_2_. Fourier transform infrared (FT-IR) spectra (Nicolet 5700, Thermo Scientific) of PEGS and PEGS-NH_2_ were recorded in the range of 600–4000 cm^− 1^.

#### Preparation of the injectable, double-crosslinked, nanocomposite adhesive hydrogels

The nanocomposite adhesive hydrogels were prepared by three steps. First, 20% (w/v) PEGS-NH_2_ and 10% (w/v) γ-PGA were dissolved in ultra-pure water. Second, Ca(NO_3_)_2_∙4H_2_O and (NH_4_)_2_HPO_4_ were added into the above solution sequentially with a constant Ca/P molar ratio (1.67:1) for in situ formation of CaP nanoparticles (ICPNs). The total inorganic/organic mass ratios were set as 10, 20 and 30%. Third, 1-ethyl-3-(3-dimethylaminopropyl)-carbodiimide hydrochloride (EDC) and N-hydroxysuccinimide (NHS) were added as coupling reagents to catalyze the reaction between the carboxyl and amino groups in the hydrogel precursors. The molar ratio of EDC to NHS was fixed at 1:1, while the molar ratio of γ-PGA (theoretical number of carboxyl groups on the γ-PGA backbone) to EDC was fixed at 15:1. The nanocomposite adhesive hydrogels were named as PEGS/xICPN, where x% denote the total inorganic/organic mass ratios. In addition, the hydrogel without CaP nanoparticles was prepared as a control group and named as PEGS. Thermogravimetric analysis (TGA; STA 449 F3, NETZSCH, Germany) was performed in the range of 25–800 °C at a rate of 10 °C min^− 1^. X-ray diffraction (XRD; 18KW/D/max2550VB/PC, Japan) were performed in the range of 15–75°. The morphologies of the completely gelled hydrogels were observed and photographed by using a scanning electron microscope (SEM; S3400-N, Hitachi, Japan).

#### Rheological studies of the nanocomposite adhesive hydrogel

A HAAKE MARS III rotational rheometer with a parallel plate (P20 TiLS, 20 mm in diameter) was used to evaluate the rheological properties of the hydrogels at 37 °C. Oscillatory time sweep tests were performed to evaluate the gelation kinetics with 5% constant strain and 10 Hz constant frequency. The gelation time was also confirmed by the vial-inversion method. Shear-thinning tests were performed to evaluate the injectability of the hydrogels with shear rates ranging from 0.2 1/s to 100 1/s. Oscillatory frequency sweep tests were performed to evaluate the stability of the completely gelled hydrogels with 5% constant strain and frequencies ranging from 0.1 to 10 Hz. All the tests were repeated three times for each group.

#### Mechanical studies of the nanocomposite adhesive hydrogels

An electronic mechanical testing machine (SANS CMT2503) equipped with a 20 N load cell was used to evaluate the tensile properties and stress relaxation properties of the hydrogels. The hydrogel precursors were casted into Teflon molds of rectangular shape (25 mm length × 10 mm width × 1.5 mm thickness). For tensile tests, the as-prepared rectangular hydrogels were stretched until fracture. For stress relaxation tests, the hydrogels were first stretched to a constant 15% tensile strain and kept still for 1200 s. All the tests were performed at a tensile rate of 50 mm min^− 1^ and repeated three times for each group.

#### Adhesive and tensile properties to bone of the nanocomposite adhesive hydrogels

To evaluate the adhesion ability to bone tissues and the tensile properties under adhesive state of the hydrogels, tibias from SD rats were prepared for adhesive and tensile teats. The tibias were wiped to remove excess liquid and treated with transverse osteotomy, and then the hydrogel precursors were applied onto the osteotomy sites. After the hydrogels were completely gelled, hydrogel-tibia constructs were formed and measured by an electronic mechanical testing machine (SANS CMT2503). All the tests were performed at a tensile rate of 1 mm min^− 1^ and repeated three times for each group.

### In vitro experiments

#### Cell culture

BMSCs were cultured in α-MEM containing 10% FBS. Cells were re-plated for expansion using trypsin when they reached 80–90% confluence. The following experiments were conducted using cells between passages 2 and 5. HUVECs were cultured in ECM, and cells were cultured at 37 °C in a humidified atmosphere of 5% CO_2_.

#### Cytocompatibility evaluation of the adhesive nanocomposite hydrogels

To determine the proliferation and viability of BMSCs cultured on PEGS/ICPN scaffolds, BMSCs (5 × 10^4^) were seeded on hydrogel surfaces for various time points. Then, CCK-8 was used to detect proliferation after 1, 3, and 5 days of culture. Cell viability was detected using CalceinAM/PI after culture for 1 day. To observe cell spreading and morphology, the cells seeded on each hydrogel were fixed with 4% paraformaldehyde and incubated with FITC phalloidin and 4′,6-diamidine-2′-phenylindole dihydrochloride (DAPI), respectively. Then, a confocal laser scanning microscopy (CLSM, Leica, Japan) was used to observe the cells, and ImageJ software (National Institutes of Health, Bethesda, MD, USA) was used to quantitatively analyze the relative cell area. Regarding the immunofluorescence assay, BMSCs (2 × 104) were seeded on different scaffolds. After culturing for 24 h, anti-vinculin antibodies were incubated overnight at 4 °C, followed by 1 h at 37 °C with a secondary antibody on fixed cells. DAPI was used for staining cellular nuclei. These samples were observed under a confocal laser scanning microscope. Each group of tests was repeated five times.

#### Determination of in vitro osteogenesis

The ability of different scaffolds to recruit BMSCs was detected using a transwell system. Briefly, cells (2 × 10^4^) were suspended in 100 μL of serum-free α-MEM and put in the upper chamber of a Transwell apparatus (Coring, USA), and different scaffolds with 200 μL of α-MEM containing 10% FBS were positioned in the lower chamber for 24 h. Then, the cells that migrated to the lower surface of the filter membrane were fixed with 4% paraformaldehyde and stained with 0.5% crystal violet. Numbers of stained cells were counted to assess migration activity. To analyze the osteogenic differentiation of BMSCs on each hydrogel, alkaline phosphatase (ALP) activity assays, ALP staining, alizarin red staining, polymerase chain reaction (PCR) with real-time quantitative analysis, and immunofluorescence were performed. In the control group, tissue culture plates were used to cultivate cells. Regarding the analysis of ALP activity, BMSCs (2 × 10^4^) were seeded on each hydrogel and cultured with an osteogenesis-induced medium (Cyagen Biosciences, USA). Detection of ALP activity was performed using the ALP test in accordance with the instructions of the manufacturer (Thermo Scientific, USA) after culture for 7 days. A BCA protein assay kit was used to measure total protein in each lysate. Then, the ALP levels were then calculated using the total amount of protein as a standard. The cells used for ALP staining were fixed in 4% paraformaldehyde and then incubated with ALP dye liquor (Beyotime) after culturing for 7 days. As for alizarin red staining, 2% alizarin red (Cyagen, China) was used to stain samples after 14 days of differentiation. Quantificationof the mineralization was conducted using 10% cetylpyridinium chloride, then the optical density (OD) measurement was made at 570 nm. Quantitative analysis of calcium contents was performed using the ortho-cresolphthalein complexone method as previously reported [20]. The expression of typical genes related to osteogenesis including runt-related transcription factor 2 (Runx2), osteocalcin (OCN), osteopontin (OPN), and Col I was confirmed using PCR after 7 days of culture, the primer sequences of each gene are listed in Table S1. Regarding the immunofluorescence assay, BMSCs (2 × 10^4^) were seeded on different scaffolds. After culturing for 24 h, anti-Runx2 and anti-OPN antibodies were incubated overnight at 4 °C, followed by 1 h at 37 °C with a secondary antibody on fixed cells. DAPI was used for staining cellular nuclei. These samples were observed under a confocal laser scanning microscope. Each group of tests was repeated five times.

#### Determination of in vitro angiogenesis

The proliferative and migratory potential of HUVECs was analyzed in the similar way as that of BMSCs, except that the HUVECs were co-cultured with BMSCs seeded on different scaffolds in a transwell system to determine the effect of PEGS/ICPN scaffolds on the interaction between BMSCs and HUVECs. Regarding the scratch wound healing assay, HUVECs were cultured to confluence in 24-well plates. Thereafter, a sterile pipette tip was used to scratch the confluent monolayer of cells. Then, the cells were co-cultured with BMSCs seeded on different scaffolds in the upper chamber of a transwell apparatus. A 6-hour and 12-hour photo was taken after scratching, and the migration area (%) was quantified using the ImageJ software. Regarding the tube formation assay, HUVECs (5 × 10^4^) were seeded in a 24-well plate with Matrigel™ (BD Biosciences, USA) covering the lower chamber. BMSCs (2 × 10^4^) were cultured on different scaffolds in the upper chamber. After 8 h of culture, calcine-AM was used to stain the polygonal structures, which was viewed under a fluorescence microscope. Quantitative analysis was performed using ImageJ software. Regarding the PCR, the RNA of vascular endothelial growth factor (VEGF), hypoxia inducible factor-1α (HIF1-α), fibroblast growth factor (FGF), and platelet endothelial cell adhesion molecule-1 (CD31) was extracted and detected in a similar manner to that used for BMSCs after 3 days of culture.

### In vivo experiments

#### Degradation and ion release of the nanocomposite hydrogels

The experiment involving animals for this study was approved by the Ethics Committee of Shanghai Jiao Tong University Affiliated Sixth People’s Hospital (Animal Committee Approval No. 2022–0434). All procedures complied with the NIH Guide for the Care and Use of Laboratory Animals. Hydrogel disks (10 mm × 2 mm, diameter × height) were used for degradation and ion release experiments. For degradation experiment, the hydrogel samples (PEGS and PEGS/20ICPN) were subcutaneously implanted into the back of the rat. On 7, 14, 21, and 28 days, the samples were collected, washed with distillated water, and dried under vacuum for 24 h at 37 °C. The initial weight and weight at the degradation time point were recorded. For ion release experiment, the samples were completely immersed in 9.0 mL PBS solution at 37.0 °C and the PBS solution was collected every week. The calcium and phosphorus ions in the solution were detected by plasma emission spectrometer (167 nm–785 nm/725, Agilent, USA).

#### Animal surgery

The tibial DO model was established using 16-week-old male SD rats, which were then randomly designated to the control, PEGS, and PEGS-20ICPN groups. The tibial DO surgery was performed as previously described [21]. In brief, the right tibia’s midshaft was exposed and a transverse osteotomy was performed following anesthesia. Then, a rail fixator (Xinzhong Medical Devices Company, China) was used to fix the distal and proximal segments of the tibia. The sterilized nanocomposite hydrogels were injected into the distraction gaps of rats belonging to the PEGS and PEGS-20ICPN groups. The DO process consisted of three consecutive phases: a five-day latency phase, a ten-day distraction phase with a distraction rate of 0.25 mm every 12 h, and a twenty-eight-day consolidation phase. The rats in three groups were sacrificed after 28 days of consolidation.

#### Micro-CT scanning and analysis

Microstructural change within the distraction site was quantitatively evaluated using amicro-CT (Scanco Medical, Bassersdorf, Switzerland). Reconstructions in three dimensions (3D) were conducted, BMD (bone mineral density) and BV/TV (bone volume ratio) of each sample were quantified with the built-in software. Also, to access the maturity level of the newly formed bone, we collected the normal tibias of SD rats and performed micro-CT scanning and quantitative analysis. Each group of tests was repeated four times.

#### Biomechanical testing

Mechanical properties of the tibia samples were evaluated through three-point bending tests within 24 h of sample collection. The tibia was posed in an anterior-posterior position while being loaded at 1 mm/min until failure. Maximum load, energy to failure, and elasticity modulus (E-modulus) were quantified after normalization to the contralateral tibia. Each group of tests was repeated five times.

#### Histological analysis

Samples were dehydrated and embedded with paraffin after complete decalcification in 15% EDTA solution. Then, they were cut into thin sections (5-μm-thick) longitudinally and stained with hematoxylin-eosin (HE) and Masson’s trichrome. Immunohistochemistry with primary antibodies against Runx2 and OCN was performed. Immunofluorescence was conducted with primary antibodies against CD31 and Hif-α. Additionally, the major organs of rats were harvested 60 days after treatment, and the HE staining was performed to assess the long-term toxicity of PEGS/ICPN hydrogels. Each group of tests was repeated at least three times.

#### Quantitative proteomics and confirmatory experiments

The detailed experimental methods of proteomics and data analysis can be found in Experimental Section, Supporting Information. Immunofluorescence staining and PCR were performed following similar procedure mentioned above to confirm the proteomic results. Expression of vinculin, α-actinin, arginase-1 (ARG-1), and inducible nitric oxide synthase (iNOS) were analyzed by immunofluorescence staining. Expression of tumor interleukin-1β (IL-1β), necrosis factor-α (TNF-α), interleukin-10 (IL-10) and transforming growth factor-β (TGF-β) were detected by PCR.

### Statistical analysis

Analysis was conducted with GraphPad Prism 8. Results were presented as mean ± SD (standard deviation). One- or two-way analysis of variance (ANOVA) with post hoc analysis determined the level of significance in the differences between groups. Statistical significance was defined as a value of *p* < 0.05 (^*^p < 0.05, ^**^p < 0.01, ^***^p < 0.001 and ^****^p < 0.0001).

## Results

### Preparation of the injectable, double-crosslinked, nanocomposite adhesive hydrogels

**Fig. 1:**
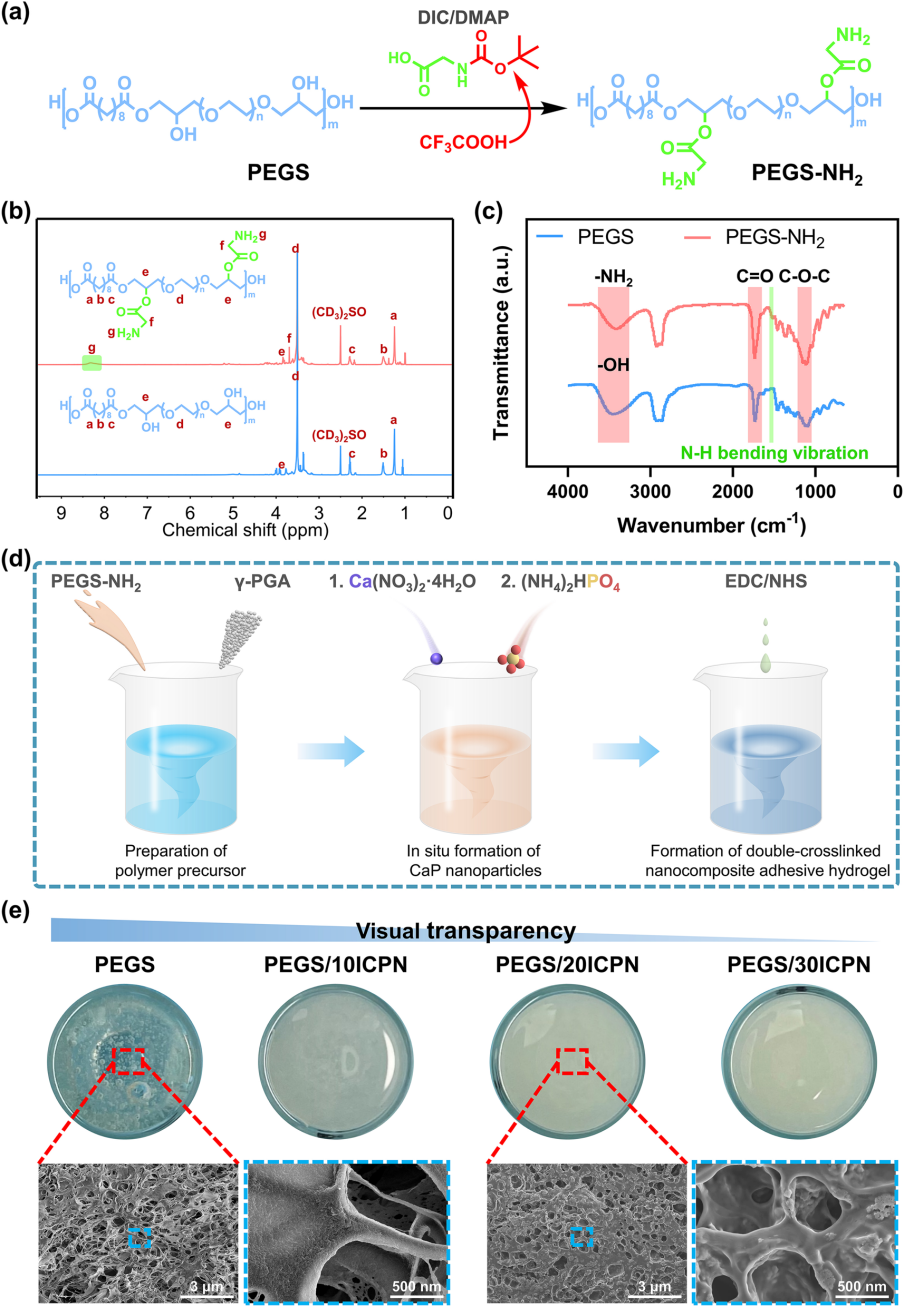
Synthesis and characterization of PEGS-NH2, and preparation and morphologies of the nanocomposite adhesive hydrogels. **a** Synthesis and molecular structure of PEGS-NH2. **b** 1H NMR spectra of PEGS and PEGS-NH2 in DMSO-d6. **c** FT-IR spectra of PEGS and PEGS-NH2 in the range of 600–4000 cm-1. **d** Schematic illustration of the preparation process of the double-crosslinked, nanocomposite adhesive hydrogels, including preparation of polymer precursor, in situ formation of CaP nanoparticles and formation of chemically crosslinked structure. **e** Macroscopic photographs and SEM images of the as-prepared hydrogels

In this study, an injectable organic-inorganic hybrid hydrogel was prepared with double-crosslinked structures based on an EDC/NHS coupling reaction and an in situ self-assembly of CaP nanoparticles. The hydrogel exhibited superior stretchability and strong adhesion to the bone. Before the preparation of the hydrogels, PEGS-NH_2_ was first synthesized via an esterification reaction followed by an acid-induced deprotection reaction (Fig. 1a, and Fig. S1, Supporting Information). As shown in Fig. 1b, compared with the proton nuclear magnetic resonance (^1^H NMR) spectrum of the original PEGS, new peaks, which were attributed to the amino group and the neighboring methylene group, appeared at 8.34 ppm (peak g) and 3.83 ppm (peak f), respectively. The amino-functionalization of PEGS was also confirmed by the Fourier transform infrared (FT-IR) spectra from the observation of a new band of the amino group-bending vibration at 1515 cm^− 1^ (Fig. 1c). All these results demonstrated the successful synthesis of PEGS-NH_2_.

The nanocomposite adhesive hydrogels were fabricated in three steps (Fig. 1d). First, the as-synthesized PEGS-NH_2_ and γ-PGA were dissolved in ultra-pure water to form a homogeneous polymer precursor. Second, Ca(NO_3_)_2_∙4H_2_O was added into the precursor to form an ionic coordination with the carboxyl group (−COO^−^-Ca^2+^) on the γ-PGA backbone, and then (NH_4_)_2_HPO_4_ was added to form CaP nanoparticles in situ between PO3–4 and Ca^2+^. The in situ γ-PGA-mediated precipitation process has been extensively used in the fabrication of nanocomposite hydrogels with uniform CaP nanoparticle dispersion [17, 18]. Meanwhile, a reversible physically crosslinked structure was established between γ-PGA and CaP nanoparticles via γ-PGA chain-CaP nanoparticle physisorption together with -COO^−^-Ca^2+^ ionic coordination [22, 23]. Third, EDC and NHS, which have been widely used as coupling reagents in the biomedical field, were added into the as-prepared organic-inorganic hybrid hydrogel precursor [24]. Under the activation and catalysis of the coupling reagents, amino groups on PEGS-NH_2_ reacted with carboxyl groups on γ-PGA to form a chemically crosslinked structure via an amination reaction. Eventually, a double-crosslinked nanocomposite adhesive hydrogel was prepared and named PEGS/ICPN. In this study, four kinds of hydrogels with different CaP contents were prepared. The actual CaP contents of these hydrogels were also measured by thermogravimetric analysis (TGA), which was consistent with the theoretical contents (Fig. S2, Supporting Information). In addition, X-ray diffraction (XRD) results confirmed the amorphous state of ICPNs in the nanocomposite hydrogels, which were beneficial for the release of PO3–4 and Ca^2+^ during the degradation of hydrogels (Fig. S3, Supporting Information). As shown in Fig. 1e, the visual transparency of the hydrogels decreased with the increase in the CaP content. The surface morphologies of PEGS and PEGS/20ICPN were further observed using a scanning electron microscope (SEM). Both PEGS and PEGS/20ICPN showed interconnected porous structures with similar pore sizes, which favored the ingrowth and proliferation of cells. The result indicated that the in situ precipitation of CaP nanoparticles with 20% CaP content would not significantly affect the morphology of the hydrogel. In addition, no obvious aggregation was observed on the surface of PEGS/20ICPN, indicating that CaP nanoparticles were uniformly dispersed in the hydrogel network. However, as the CaP content increased to 30%, the interconnected porous structure almost disappeared, and CaP nanoparticles even appear to aggregate due to the high inorganic content (Fig. S4, Supporting Information). To further study the effects of compositions and structures on mechanical and biological properties, all four adhesive hydrogels were prepared for subsequent tests.

### Injectability, stretchability and bone adhesion ability of the nanocomposite adhesive hydrogels

**Fig. 2:**
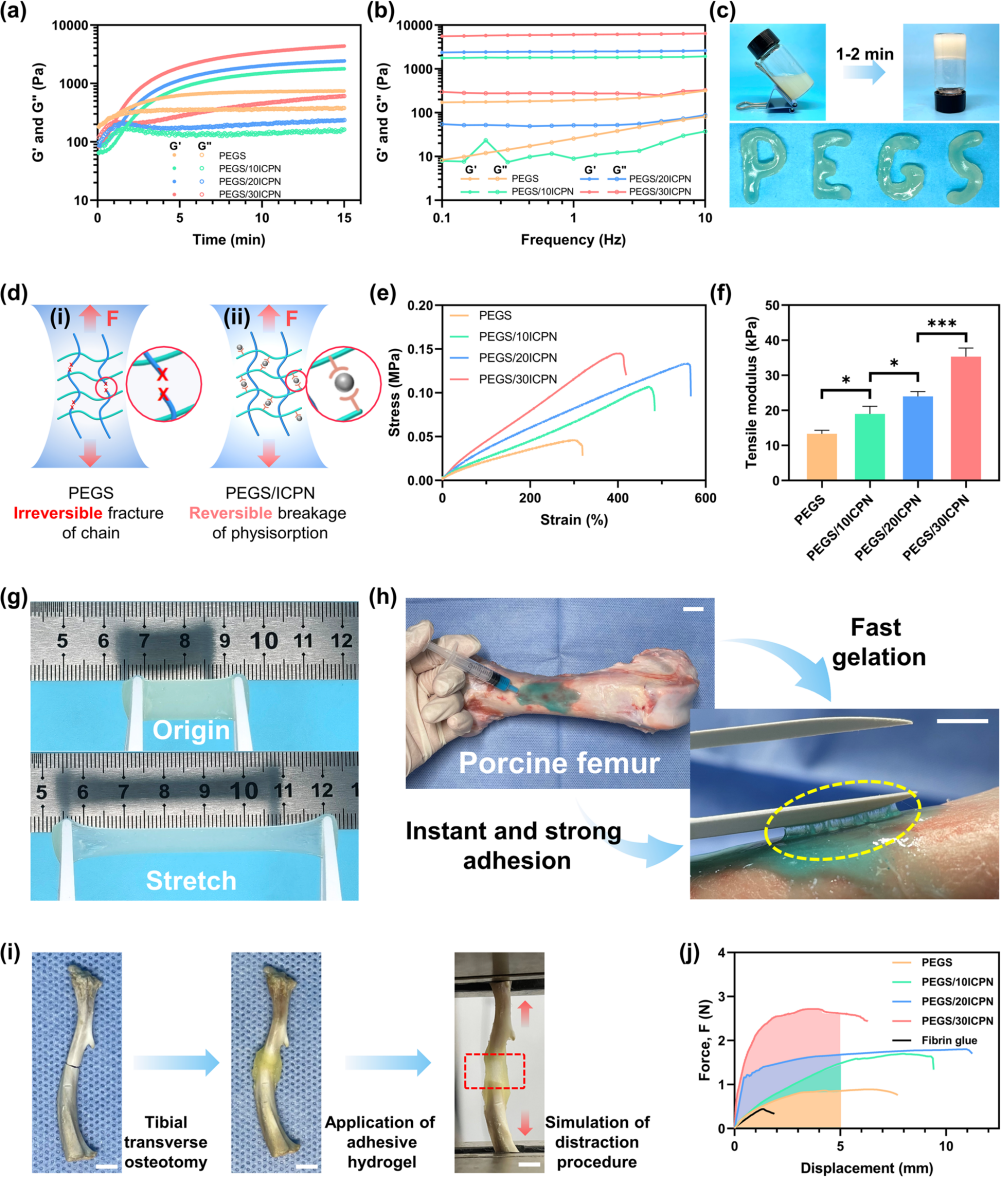
Rheological, mechanical and adhesive properties of the adhesive hydrogels. **a** Oscillatory time-sweep rheological analysis was performed to monitor the gelation process of the hydrogels. **b** Oscillatory frequency-sweep rheological analysis was performed to evaluate the stability of the completely gelled hydrogels in the range of 0.1–10 Hz. **c** Photographs of the gelation process exhibited by vial-inversion method and letters “PEGS” written by the hydrogels. **d** Schematic illustrations of the hydrogels without (i) and with (ii) CaP nanoparticles under stretched state. **e** Ultimate tensile stress-strain curves and **f** tensile modulus of the hydrogels. **g** Photographs of the rectangular PEGS/20ICPN hydrogel in the original and stretched states. **h** Photographs illustrating the injectability, fast gelation, instant and strong adhesion to bone of PEGS/20ICPN hydrogel (scar bar: 10 mm). The hydrogel was stained with Reactive blue (Macklin, Shanghai). **i** Photographs illustrating the in vitro simulation of the distraction procedure, including tibial transverse osteotomy, application of hydrogels and tensile tests (scar bar: 5 mm). **j** Typical force-displacement curves of the hydrogels and commercial fibrin glue adhered to bones

The injectability of adhesive hydrogels is essential for applying in the DO procedure. To evaluate the gelation kinetics of the hydrogels with different inorganic contents, oscillatory time sweep tests were performed at physiological temperature (37 °C). Figure 2a shows that the storage moduli (*G′*) and loss moduli (*G″*) of each hydrogel possessed an intersection point in the range of 1–2 min, indicating that the hybrid hydrogel precursors could form hydrogels in the presence of coupling reagents. The appropriate gelation time further endowed the adhesive hydrogels with ideal injectability and manipulability, which were suitable for clinical practices in DO procedures. In addition, shear-thinning properties of the hydrogels were also confirmed, and a minimum 16-gauge needle were used for injection in practice (Fig. S5, Supporting Information). To further evaluate the mechanical stability of the completely gelled hydrogels, oscillatory frequency sweep tests were conducted. As shown in Fig. 2b, the *G′* of all hydrogels were consistently higher than the corresponding *G″*, indicating that the adhesive hydrogels were mechanically stable. In addition, the gelation time and injectability were also confirmed using the vial-reversion method and injection molding (Fig. 2c).

Since the adhesive hydrogel would be stretched on the osteotomy site, the superior stretchability of bulk hydrogel plays an essential role in the application of the DO procedure. Figure 2d illustrates two different breakage mechanisms of the hydrogels network under the stretched state. The PEGS hydrogel without ICPNs occurred irreversible fracture of the chain, while the PEGS/ICPN hydrogel with ICPNs underwent the reversible breakage of γ-PGA chain-CaP nanoparticle physisorption via the intermediation of the -COO^−^-Ca^2+^ ionic coordination [22, 23]. As shown in Fig. 2e, the PEGS/ICPN hydrogels exhibited superior stretchability to the PEGS hydrogel, indicating that the hydrogels with ICPNs were able to withstand higher external force and larger deformation due to the reversible breakage mechanism. The reversible breakage and reformation of γ-PGA-CaP physisorption endow the nanocomposite adhesive hydrogel with effective energy dissipation properties. Notably, the PEGS/30ICPN hydrogel exhibited less elongation at break, probably due to the undesirable aggregation of CaP nanoparticles and the resulting heterogeneous bulk hydrogel. The tensile moduli of the adhesive hydrogels were further calculated from the ultimate tensile stress-strain curves. As shown in Fig. 2f, the tensile moduli increased with the increase in the CaP content. Figure 2g shows the superior stretchability of the PEGS/20ICPN hydrogel. Additionally, the stress relaxation behaviors of hydrogels were also investigated (Fig. S6, Supporting Information). Compared with the hydrogels without ICPNs, all PEGS/ICPN hydrogels showed significant stress relaxation under a constant tensile strain. With the increase of ICPNs content, the hydrogels showed faster stress relaxation behavior due to the more efficient energy dissipation mediated by the breakage of γ-PGA-CaP physisorption. Notably, the PEGS/30ICPN hydrogel exhibited an unusual stress relaxation rate, presumably due to the heterogeneous dispersion of ICPNs.

In addition to the superior stretchability, the adhesion ability of hydrogels to bone is equally crucial. As shown in Fig. 2h, the blue-stained PEGS/20ICPN hydrogel was first injected into the porcine femur, then the hydrogel gelled fast on the bone surface and exhibited instant and strong adhesion to bone tissues. The observed phenomenon was achieved by the multiple adhesion mechanisms, including covalent amide bonds and -COO^−^-Ca^2+^ ionic coordination formed between the hydrogel and bone. To further confirm the clinical practicality of the adhesive hydrogels in the DO procedure, in vitro adhesive and tensile tests inspired by an in vivo distraction process were conducted. Figure 2i illustrates the process of in vitro adhesive and tensile tests. First, the tibia obtained from a SD rat was treated with transverse osteotomy. Then, the adhesive hydrogel was applied to the osteotomy site. After the hydrogel was completely gelled, the as-formed hydrogel-tibia construct was stretched and tested. As shown in Fig. 2j, compared with commercial fibrin glue, the experimental hydrogels exhibited robust adhesion to bone and were stretched by over 5 mm without interfacial detachment and bulk rupture, indicating that all the adhesive hydrogels satisfied the basic mechanical requirement of an animal experiment. Moreover, the PEGS/20ICPN hydrogel showed the optimal stretchability under adhesive state, which was consistent with the results of tensile tests.

### In vitro viability, adhesion, proliferation and pro-osteogenic effects of the nanocomposite adhesive hydrogels

**Fig. 3:**
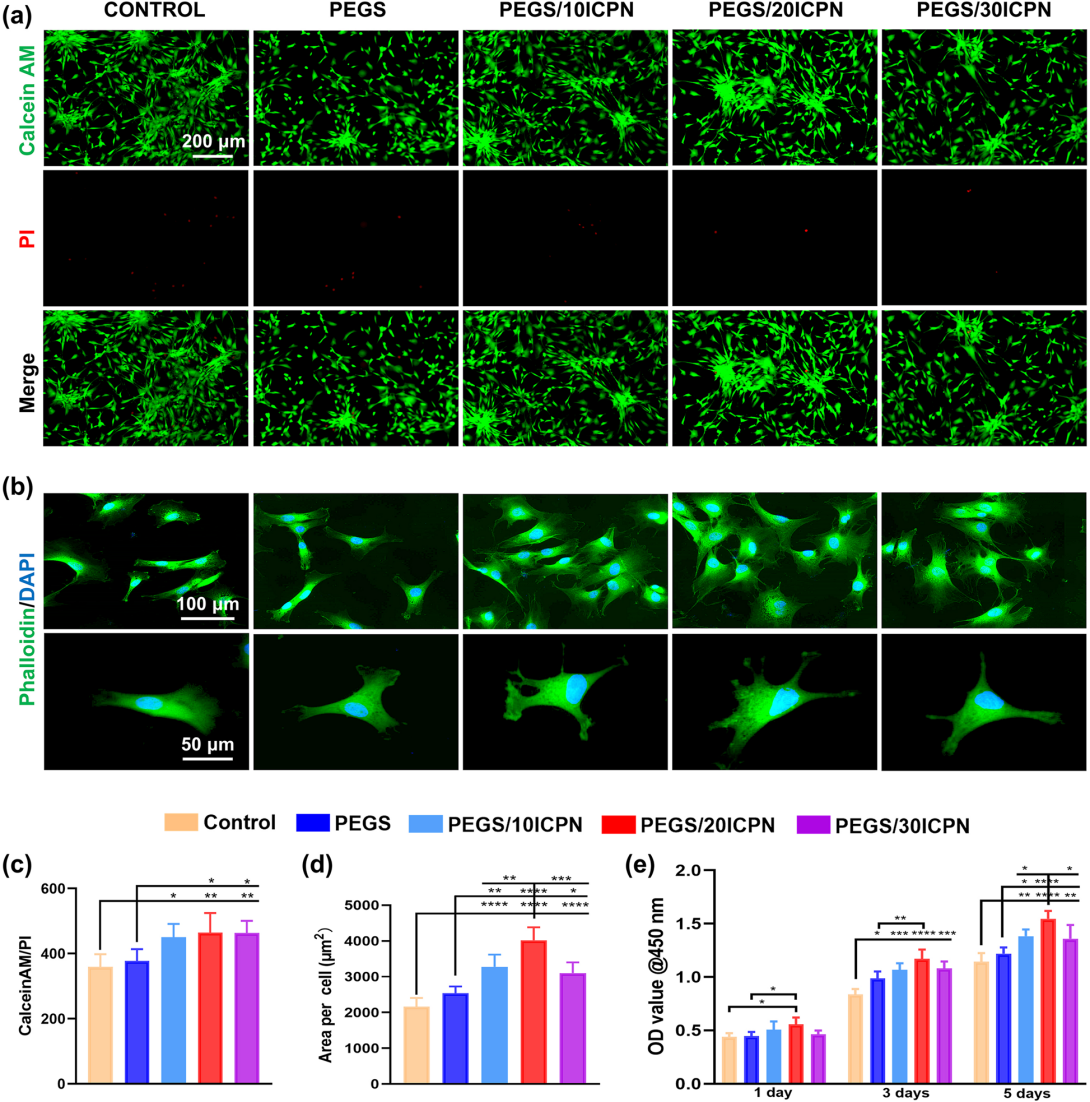
Viability, adherence, and proliferation of the nanocomposite adhesive (BMSCs as a cell model). **a** Live/dead staining of BMSCs cultured on each hydrogel surface for 24 h. **b** Phalloidin (green) and DAPI (blue) staining exhibit the morphology of BMSCs growing on the composite hydrogels after 3 days. **c** Quantification of live/dead assay. **d** Quantification of cell spreading area. **e** CCK8 assay for 1, 3 and 5 days of culture

BMSCs were cultured on each hydrogel to evaluate the biocompatibility of PEGS/ICPN hydrogels. The cell toxicity of PEGS/ICPN scaffolds was tested by staining BMSCs with Calcein-AM/PI. A very small number of dead cells were observed on the surface of each hydrogel after 24 h, indicating that there was no cytotoxic effect induced by any scaffold (Fig. 3a). Also, the quantitative analysis of live/dead assays showed that the cell viability of BMSCs on the PEGS/ICPNs was significantly higher compared to the control and PEGS groups (Fig. 3c).

In Fig. 3b, FITC (fluorescein isothiocyanate)-phalloidin staining demonstrated that F-actin, a crucial skeleton component of cells, appeared more in BMSCs on PEGS/ICPN scaffolds in comparison with the others, indicating the superior cell adhesion-enhancing properties of PEGS/ICPN hydrogels. To identify the underlining mechanisms, fluorescent staining was performed to investigate the expression of vinculin (Fig. S7, Supporting Information). The results revealed that PEGS/ICPN hydrogels showed more intense expression than PEGS and control group, and PEGS/20ICPN expressed vinculin maximally. Furthermore, BMSCs cultivated on PEGS/ICPN presented a more spread and stretched morphology in comparison with those in the control and PEGS groups (Fig. 3d), indicating the porous surface and mechanical properties of PEGS/ICPN hydrogels were favorable for cell adhesion.

The cell proliferative potential of BMSCs on each scaffold was assessed using the CCK-8 assay on days 1, 3, and 5 (Fig. 3e). The BMSCs on PEGS/ICPN scaffolds exhibited stronger proliferative activity on days 1 and 3. On day 5, the proliferation of BMSCs on the PEGS/20ICPN was significantly higher in comparison with all the other groups. The above results revealed that all scaffolds were highly cytocompatible, among which the nanocomposite hydrogels containing 20% CaP NPs (PEGS/20ICPN) provided the most preferable conditions for cell viability, adhesion and proliferation, providing the foundations for tissue regeneration.

**Fig. 4:**
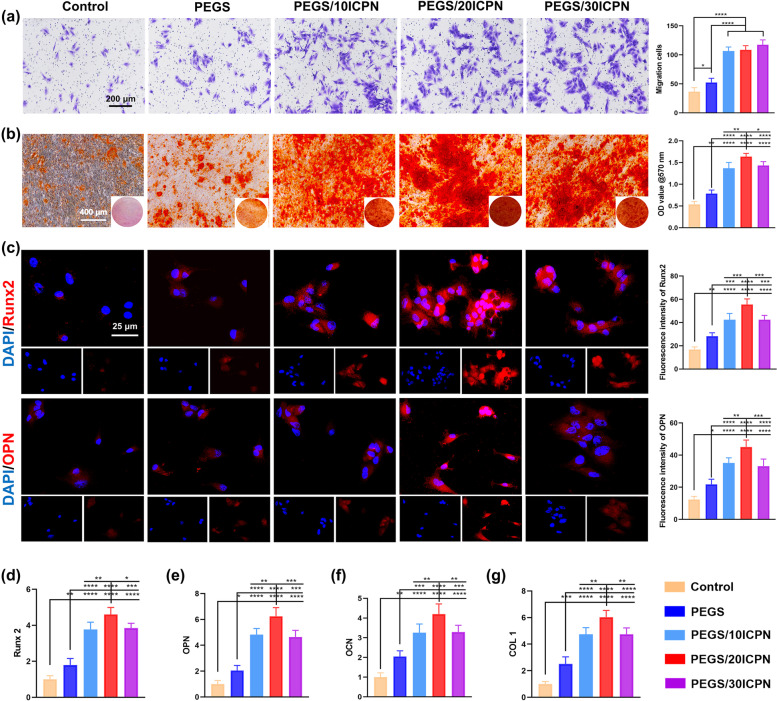
In Vitro pro-osteogenic effects of the PEGS/ICPN hydrogels. **a** BMSCs migration detection via Transwell assays. **b** Alizarin red staining images and the quantitative analysis after 14 days of culture. **c** Runx2 and OPN immunofluorescent staining of BMSCs cultured for 7 days. Runx2 and OPN were stained red and the nuclei was stained blue. **d**-**g** PCR results of osteogenesis-associated gene expression including Runx 2, OPN, OCN, and Col 1

The migration capacity of BMSCs promoted by PEGS/ICPN scaffolds was evaluated using the Transwell assay. We found that the PEGS/ICPN groups contained more migrated cells than the control and PEGS groups (Fig. 4a). However, no significant difference was found among the three PEGS/ICPN groups with different mass ratios of ICPNs. The possible reason is that the cells cultured in the Transwell system were not directly seeded on the hydrogels, their migratory behavior was only affected by the release of calcium and phosphate ions when the composite hydrogels degraded. Then, the best osteogenesis conditions were determined by different experiments performed using cells cultured on different samples. The ALP staining (Fig. S8, Supporting Information) and alizarin red staining (Fig. 4b) revealed that PEGS/ICPN groups all showed higher staining compared to the control and PEGS groups, and the PEGS/20ICPN group, in particular, presented the highest staining. The quantitative detection calcium contents (Fig. S9, Supporting Information) and the ALP activity assay (Fig. S10, Supporting Information) confirmed this observation. To further confirm the above results, the gene expression levels associated with osteogenesis including Runx2, OPN, OCN, and Col 1 were tested with immunofluorescence staining (Fig. 4c) and PCR (Fig. 4d-g). Both the quantitative immunofluorescence analysis and PCR results indicated that the BMSCs in the PEGS/ICPN groups showed noticeably higher gene expression compared to the other groups, suggesting that the introduction of CaP NPs could create a more advantageous osteogenic environment. Specifically, PEGS/20ICPN presented the most optimal osteogenic differentiation results, probably due to the more uniform distribution of CaP NPs.

### In vitro pro-angiogenic effects of the nanocomposite adhesive hydrogels

**Fig. 5:**
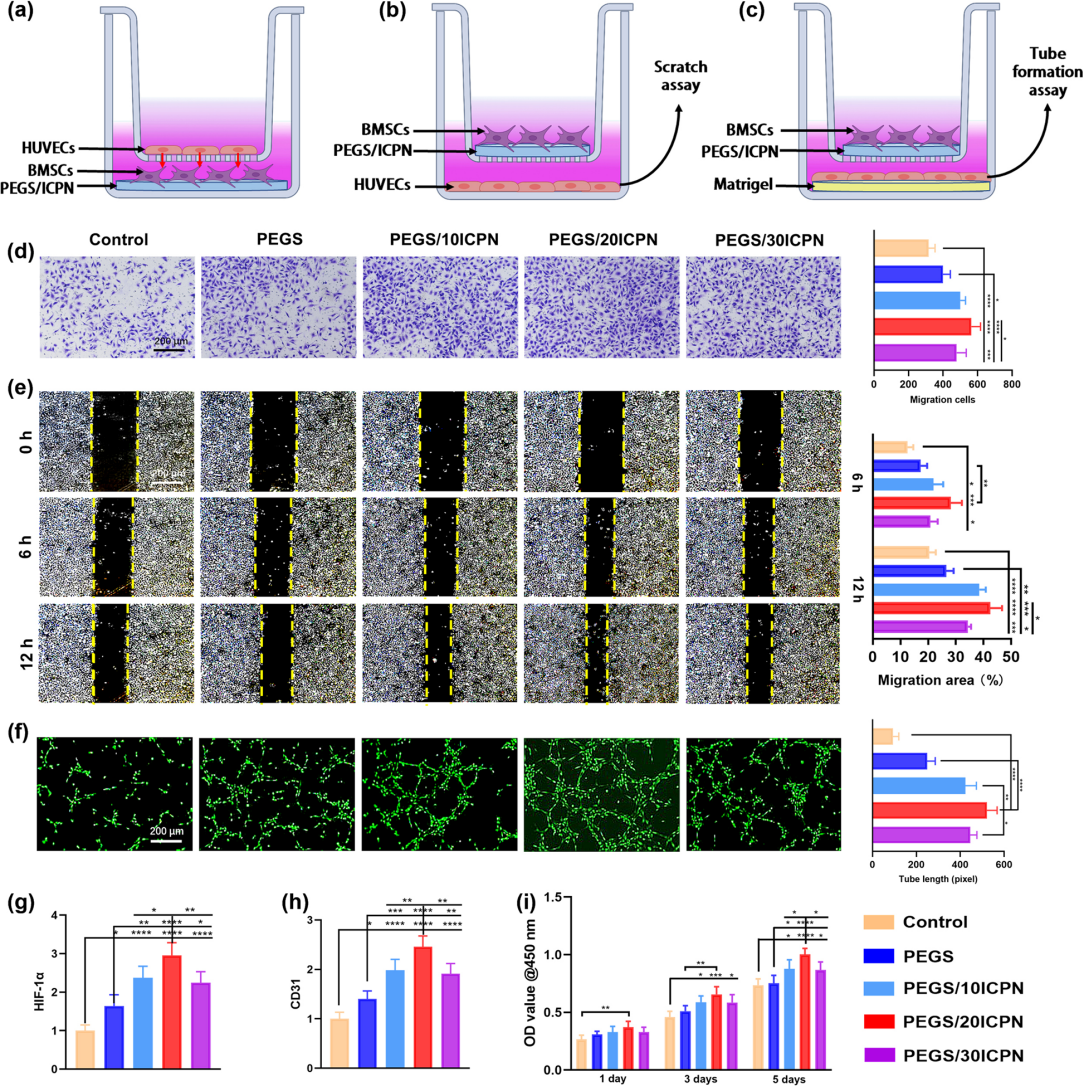
In Vitro pro-angiogenic effects of the nanocomposite hydrogels. **a**-**c** Experimental schematic of transwell assay, scratch wound assay and tube formation assay. **d** Transwell assay for 24 h. **e** Scratch wound assay after 6 h and 12 h. **f** Immunofluorescence of tube formation. **g**, **h** Angiogenesis related gene expression (HIF-1α and CD31). **i** Proliferation of HUVECs

The transwell system was used to study the effects of PEGS/ICPN hydrogels on the interaction between BMSCs and human umbilical vein endothelial cells (HUVECs) (Fig. 5a-c). The migratory behavior of HUVECs enhanced by BMSCs cultured on PEGS/ICPN hydrogels were assessed using the transwell assay and scratch wound healing assay. We observed that the PEGS/20ICPN group exhibited a higher number of migrated cells and a faster healing rate than the other groups (Fig. 5d, e). Moreover, incubation of HUVECs with scaffold-mediated BMSCs in the PEGS/20ICPN group showed significantly longer capillary-like structures than in the other groups, as demonstrated by the tube formation assay at 5 h (Fig. 5f). Given that angiogenesis-related genes including HIF1-α, VEGF, CD31, and FGF are highly expressed and considered to be crucial during the DO process, their levels were determined by PCR. The results shown in Fig. 5g, h and Fig. S11, Supporting Information indicated that PEGS/ICPNs promoted angiogenesis at the gene level, and the highest expression was found in the PEGS/20ICPN group. In addition, the proliferative potential HUVECs was evaluated by the CCK8 assay. HUVECs proliferation was significantly promoted by the PEGS/ICPN scaffolds, among which the PEGS/20ICPN scaffold exhibited more significant promoting effects (Fig. 5i). These results suggest that BMSCs mediated by PEGS/ICPN scaffolds triggered a strong angiogenic response of HUVECs via positively enhancing the interactions between BMSCs and HUVECs [25].

### Degradation and ion release of the nanocomposite hydrogels

The in vivo weight loss of PEGS hydrogel is significantly higher than that of the PEGS/20ICPN hydrogel after 7, 14, 21, and 28 days of implantation (Fig. S12, Supporting Information). The results of ion release experiments illustrated that PEGS/20ICPN hydrogel allowed for a sustainable and continuous release of calcium and phosphate into the local microenvironment, and no burst release was observed (Fig. S12, Supporting Information).

### In vivo pro-osteogenic and pro-angiogenic effects of the nanocomposite adhesive hydrogels during DO

**Fig. 6:**
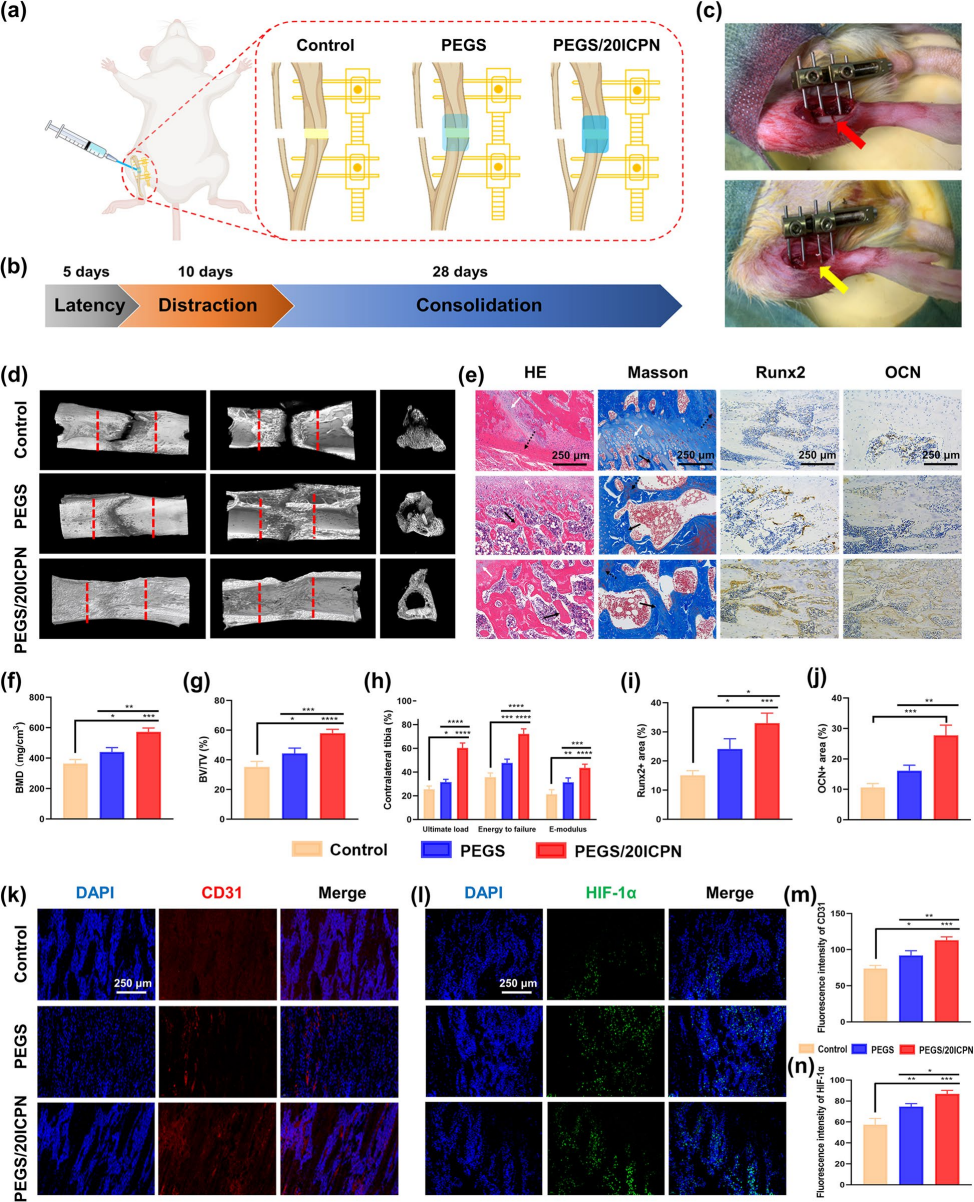
PEGS/20ICPN hydrogel improved both bone regeneration and vascularization in the regeneration zone. **a** Grouping scheme of rat tibial DO model. **b** Timeline for distraction protocol. **c** Application of external fixation and tibial osteotomy during surgery. Red arrow, osteotomy. Yellow arrow, application of PEGS/20ICPN hydrogel. **d** Representative 3D images of the distraction zone after consolidation for 4 weeks, indicating more continuous callus in the PEGS/20ICPN group. Red dotted lines illustrate the regeneration zone in between. **e** Representative images of HE, Masson, and immunohistochemical staining of RUNX2 and OCN. White arrows, cartilaginous tissue. Dotted arrows, fibrous-like tissue. Black arrows, newly regenerated trabecular bone. **f**, **g** Analysis of BMD and BV/TV of the new bone. **h** Mechanical properties including ultimate load, energy to failure and modulus of elasticity (E-modulus) of the regenerates. **i**, **j** Quantitative analysis of the immunohistochemical staining in (**e**). **k**, **l** Immunofluorescence images of CD31 and HIF-1α for the regeneration zone. **m**, **n** Quantitative analysis of the immunofluorescent staining in (**k**, **l**)

The grouping scheme and treatment timeline for the distraction protocol are shown in Fig. 6a, b. Representative photographs of the rat DO model and the application of the composite hydrogels during surgery are shown in Fig. 6c. Based on the results of mechanical tests and in vitro cell biology evaluation, PEGS/20ICPN hydrogel showed superior bone adhesion, stretchability, and pro-osteogenic and pro-angiogenic effects, which was expected to be the optimal choice to serve as artificial periosteum and accelerate the dynamic DO process. The in vivo effects of PEGS/20ICPN were compared with PEGS group and blank control group.

Following consolidation for 4 weeks, the 3D reconstruction images revealed that there were obvious defect gaps located in the middle of the regenerated in the control group, while a significant amount of new bone was formed in the PEGS/20ICPN group (Fig. 6d). Furthermore, quantitative analysis of the distraction gaps revealed that significantly higher BMD and BV/TV values were found in the PEGS and PEGS/20ICPN groups than those in the control group, with the PEGS/20ICPN group exhibiting the most optimal pro-osteogenic ability in vivo (Fig. 6f, g). The BMD values of the Control, PEGS, PEGS/20ICPN groups respectively reached 51.21, 61.89, and 80.48% of the normal value. And BV/TV values respectively reached 53.04, 66.58, and 87.25% of the normal value. The results of mechanical testing showed that the PEGS/20ICPN group achieved a significant enhancement in ultimate load, energy to failure, and E-modulus when compared with the other two groups after being normalized with the contralateral tibia (Fig. 6h).

Regarding histological assessment, HE and Masson’s trichrome staining exhibited various amounts of fibrous-like tissue, cartilaginous tissue, and newly regenerated bone, parallel to the distraction forces (Fig. 6e). However, more mature trabecular bone and less cartilaginous or fibrous-like tissues were observed in the PEGS/20ICPN group compared to the other two groups at 4 weeks. Furthermore, immunohistochemical analysis of the distraction gaps demonstrated more intense staining of Runx2 and OCN in the PEGS/20ICPN group than in the control group (Fig. 6i, j).

Neo-angiogenesis induced by the scaffolds could supply sufficient nutrients to promote bone reconstruction during DO. CD31 is considered an essential biomarker of endothelial cells and plays a crucial role in cell adhesion [26]. Immunofluorescence staining of CD31 showed that the PEGS and PEGS/20ICPN groups exhibited significantly more newly formed blood vessels than the control group **(**Fig. 6k**)**. In addition, the PEGS/20ICPN group presented the most intensive expression, suggesting the most mature formation of vessels **(**Fig. 6m). HIF-1α, an elemental transcription factor expressed in hypoxic condition, triggers the expression of genes connected with angiogenesis (VEGF), metabolism (ALDA, PGK1, ENO1, PFKL, LDHA), and erythropoiesis (EPO) [27]. The sustained expression of HIF-1ɑ is crucial for maintaining chondrogenic/osteogenic balance in endochondral ossification [28]. In our previous study, we found that PEGS-based composite hydrogels could induce ahypoxia-mimicking environment and activate the HIF-1α signaling pathway, consequently promoting the progress of endochondral ossification [29]. In present study, immunofluorescence test of the distraction gap demonstrated the most intensive staining of HIF-1α in the PEGS/20ICPN group (Fig. 6l, n), which is in consistence with the the in vitro study. These results indicate that exerting synergistic pro-angiogenic and pro-osteogenic effects is another critical mechanism by which PEGS/20ICPN enhances bone formation in DO, aside from its direct promotion of osteogenesis.

### Proteomic analysis of the distraction callus induced by PEGS/20ICPN

**Fig. 7:**
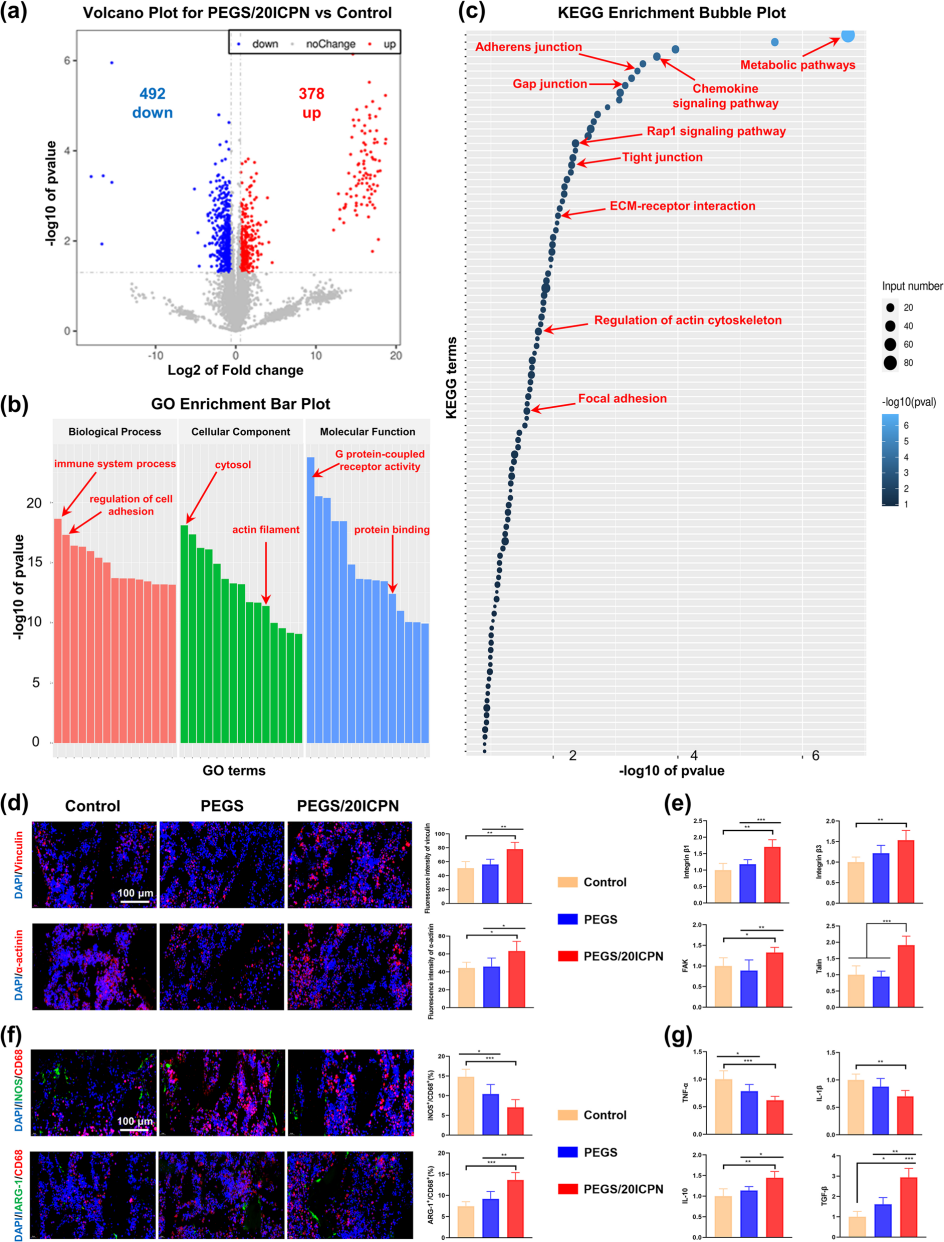
Proteomic and bioinformatic analysis across the PEGS/20ICPN hydrogel. **a** Volcano plot of the DEPs between groups. Blue points represent down-regulated proteins. Red points represent upregulated proteins. **b**, **c** GO and KEGG protein enrichment analysis. **d** Immunofluorescence staining of the newly formed bone with DAPI (blue), Vinculin (red), and α-actinin (red). **e** Relative mRNA levels of genes related to cell adhesion in all groups. **f** Immunofluorescence staining of the newly formed bone with DAPI (blue, nuclei), CD68 (red, a marker of macrophages), iNOS (green, a marker of M1), and ARG-1 (green, a marker of M2). Quantitation of the rate of iNOS+/CD68+ macrophages and ARG-1+ / CD68+ macrophages. **g** Relative mRNA levels of TNF-α, IL-1β, IL-10, and TGF-β of the newly formed bone in all groups

To further investigate the underlying mechanism, we performed proteomic analysis on the distraction callus induced by PEGS/20ICPN, and the group with no intervention of materials was set as a control. Volcano plot analysis of differentially expressed proteins (DEPs) for PEGS/20ICPN vs control showed that 492 genes were downregulated, and 378 genes were upregulated (Fig. 7a). Gene ontology (GO) terms shown in Fig. 7b indicated that the effects of the CaP nanocomposite hydrogel on the biological process, cellular component, and molecular function of the newly formed bone mainly focused on the immune response, regulation of cell adhesion, actin filament, G protein-coupled receptor activity, and protein binding. Kyoto Encyclopedia of Genes and Genomes (KEGG) pathway analysis was also performed to detect the most enriched signal transduction pathways of these DEPs. The results revealed that the PEGS/20ICPN hydrogel could significantly upregulate adherence-related pathways, such as adherens junction, gap junction, Rap1 signaling pathway, tight junction, ECM-receptor interaction, regulation of actin cytoskeleton, and focal adhesion (Fig. 7c). In addition, an immunoregulation-related pathway including the chemokine signaling pathway was also significantly upregulated in the PEGS/20ICPN group.

To verify the cell adhesion signaling pathway which the PEGS/ICPN hydrogel might activate, level of genes related to focal adhesion kinase (FAK) pathway, such as Vinculin, α-actinin, Integrin β1, Integrin β3, FAK, and Talin were detected by immunofluorescence staining (Fig. 7d) or PCR (Fig. 7e). The results demonstrated that all the above-mentioned genes were upregulated in the newly formed bone induced by PEGS/20ICPN, which was consistent with the proteomic analysis.

To confirm our proteomic results that PEGS/20ICPN promoted the DO process by modulating immune reaction, immunofluorescence staining of the distraction callus was performed to determine the M1-M2 transition of macrophages. As expected, the results suggested the application of PEGS/20ICPN significantly reduced the M1 polarization rate (iNOS1/CD68) and increased the M2 polarization rate (ARG-1/CD68) (Fig. 7f). The expression of polarization-related genes including TNF-α, IL-1β, IL-10 and TGF-β was determined by PCR. The results indicated that PEGS and PEGS/20ICPN reduced the levels of TNF-α and IL-1β genes in the distraction callus. Regarding the anti-inflammatory effects, PEGS and PEGS/20ICPN could significantly increase the levels of IL-10 and TGF-β genes. Among them, the PEGS/20ICPN exhibited the most outstanding anti-inflammatory effects (Fig. 7g).

### In vivo assessment of long-term systemic toxicity

As biosafety is another critical criterion in the medical applications of biomaterials, the long-term systemic toxicity of PEGS/ICPN hydrogels in vivo was assessed. No noticeable changes in the overall health or behaviors of the rats were observed during the treatment period (up to 60 days after surgery). The HE staining showed that the application of PEGS/20ICPN hydrogel for DO did not cause any harm to the major organs including the heart, liver, spleen, lung, and kidney (Fig. S13, Supporting Information), suggesting that the nanocomposite hydrogel could effectively promote the DO process and has excellent biocompatibility and biosafety.

## Discussion

The technique of distraction osteogenesis is one of the most commonly used methods to reconstruct segmental bone defect in clinic. However, patients have to suffer an unwanted lengthy period of time wearing the burdensome external fixator due to the slow formation and calcification of new bone, during which a high rate of complications may occur. In the course of DO treatment, the periosteum under mechanical stress is the main source of newly formed bone. Damage or absence of periosteum commonly leads to poor bone regeneration and further delays the treatment time of DO, which might be efficiently improved by application of a bionic periosteum. Therefore, inspired by the dynamic DO process and the mechanical stimulation transferred by a natural periosteum, we design a nanocomposite organic−inorganic hydrogel bionic periosteum with multiple biological functions. This bionic periosteum is featured with excellent injectability for convenient application, robust bone adhesion and superior stretchability for matching the DO process and transferring mechanical stress to the underneath new bone, and enhanced osteogenic activity for accelerating new bone formation and calcification.

The components, composition and structure of the bionic periosteum established the foundation for its physicochemical and biological characteristics. The newly designed bionic periosteum developed in this study was based on the matrix of a poly(γ-glutamic acid)-crosslinked amino-functionalized PEGylated poly(glycerol sebacate) (γ-PGA/PEGS-NH2) adhesive hydrogel, which was once designed as an effective adhesives for joint wound treatment under dynamic physiological conditions [16]. Nonetheless, this adhesive hydrogel remained limited by their insufficient stretchability and poor osteogenic activity for application to DO process. Calcium phosphate (CaP) has been widely incorporated in fabricating organic-inorganic hybrid hydrogels to enhance their mechanical and osteogenic properties, as CaP promotes hydroxyapatite mineralization and new bone calcification [30–34]. However, direct addition of the CaP component into the polymer system often severely hindered the biomedical application of the hybrid hydrogel due to the existence of two-phase interface incompatibility, which could lead to adverse effects on both biological and physical properties [35, 36]. To solve this problem, γ-PGA bearing abundant carboxyl groups was introduced to induce the in situ precipitation of CaP nanoparticles via -COO^−^-Ca^2+^ ionic coordination. Then, the double-crosslinked structures constructed based on γ-PGA chain-CaP nanoparticle physisorption and EDC/NHS coupling reaction effectively enhanced stretchability of the nanocomposite hydrogel. Moreover, during the injection and gelation process of hydrogel precursor on osteotomy site, the nanocomposite hydrogel can form covalent amide bonds and -COO--Ca^2+^ ionic coordination with the carboxyl and amino groups and Ca^2+^ on the surface of bone, which collectively contribute to the robust adhesion to bone tissue. Additionally, this bionic periosteum was expected to exhibit robust pro-osteogenic activity by introduction of in situ precipitated and evenly distributed CaP NPs.

It is well recognized that both chemical and physical cues of biomaterials such as chemical components and surface properties can profoundly influence the cellular behavior and determine the fate of stem cells [37, 38]. In this study, PEGS/ICPN scaffolds provide preferable conditions for viability, adhesion, proliferation, and osteogenic differentiation of BMSCs. Consistent with our findings, the results of another investigation exhibited the lowest cytotoxicity on BMSCs cultured on the hydrogel-based matrix enriched with CaP nanoparticles [25]. It also was proved that an appropriate nanoscale disorder surface or nano displaced topography could significantly enhance cellular adhesion, proliferation, and differentiation compared with a planar surface [39]. And a rapid stress relaxation matrix could invoke BMSCs shape changes, proliferation, and osteogenic differentiation and subsequently promote the bone matrix formation [40]. Additionally, we believed that the release of calcium and phosphate ions during the process of degradation also played a positive role in promoting BMSCs migration, adhesion, proliferation, and differentiation. In a study that aimed to investigate the individual impacts of calcium and phosphate ions released from composite materials on BMSCs, the investigators observed that calcium could promote cell proliferation while both calcium and phosphate ions enhanced alkaline phosphatase production and mineralization. The PCR analysis in the study also revealed positive effects of calcium and phosphate ions on the expression of osteogenic-related markers [41]. Therefore, it is reasonable to hypothesize that the enhanced adhesion and osteogenic differentiation of BMSCs induced by PEGS/ICPN should be ascribed to the changed surface nano-topography, improved mechanical properties, and release of calcium and phosphate ions [39, 41].

Hydroxyapatite (HA) nanoparticles, which is mainly comprised of calcium phosphate (CaP), has been previously proven to have pro-angiogenic potential. Specifically, He et al. demonstrated that HA-incorporating composite scaffolds seeded with BMSCs promoted the expression of angiogenic-related genes to a significantly higher degree than scaffolds without HA, leading to a more vigorous angiogenesis and osteogenesis [42]. In current study, we observed that BMSCs mediated by PEGS/ICPN scaffolds triggered a strong angiogenic response of HUVECs in vitro, suggesting that the nanocomposite hydrogels were able to enhance the interactions between BMSCs and HUVECs. It is well known that the process of new bone formation during DO is reported to be closely coupled with robust angiogenesis [43]. Impairment of angiogenesis caused by aging or radiation reportedly inhibits new bone regeneration during DO [44, 45]. On the contrary, enhancement of angiogenesis with various cytokines or stem cells has reportedly shown positive effects in accelerating new bone formation and consolidation in animal DO models [46]. Based on the above rationale, we believed that PEGS/ICPN scaffolds could promote new bone formation and mineralization during DO through the synergistic effects of angiogenesis and osteogenesis.

A controllable biodegradation performance of hydrogels is of great importance during bone regeneration. The in vivo degradation test showed that the introduction of in situ precipitated CaP nanoparticles could reduce the degradation rate of PEGS hydrogel, which might indicate that the inorganic/organic double crosslinked method could enhance the structural stability of hydrogel in comparison with the simple physical mixing. The nano composite hydrogels also enabled a sustainable delivery of bioactive ions (calcium, phosphate) into the surrounding microenvironment, which is advantageous for cell recruitment, adhesion, migration, and differentiation for tissue regeneration.

To better verify the potential of PEGS/ICPN hydrogels for imitating the mechano-transduction process of natural periosteum, the DO process is expected to be the most suitable animal experimental model due to the dynamic distraction process can exert tension force on the osteotomy site through the hydrogel strongly adhering to bone surface and stimulate new bone formation. For the control group, there were obvious defect gaps and more fibrous-like tissues located in the middle of the regenerated due to the absence of periosteum and the lack of a barrier to surrounding soft tissue. While more mature trabecular bone and less cartilaginous or fibrous-like tissues were observed in the PEGS/20ICPN group. Moreover, blood supply reconstruction was also observed to be promoted by the PEGS/20ICPN hydrogel. Finally, proteomic analysis of the newly formed bone was performed to explore the underlying mechanism, and the regulation of cell adhesion and immune process was found to be the essential mechanism.

Cell adhesion, the initial stage of reciprocal action between materials and cells, directly exerts regulations on cellular behaviors [47]. It is now well documented that cell adhesion and subsequential contractile cytoskeletal organization is crucial in both osteogenesis and angiogenesis [48]. Focal adhesion kinase (FAK), an adhesion-related signaling, has been confirmed to be fundamental to osteogenesis stimulated by cell–material interactions via promoting integrin-mediated cell adhesions, survival, proliferation, and migration [49]. In this study, it was revealed through proteomic analysis that FAK signaling pathway was also activated by PEGS/ICPN hydrogel.

Recently, investigations into the immune response in biomaterial-mediated osteogenesis have increased. Macrophages, which can be polarized into pro-inflammatory phenotype (M1) or an anti-inflammatory phenotype (M2) under specific circumstances, are one of the most crucial immune cells responsible for implanted biomaterials and subsequent bone regenerative processes [50]. M1 phenotype secret many pro-inflammatory factors, including inducible nitric oxide synthase (iNOS), tumor necrosis factor- a (TNF-a) and interleukin-1β (IL-1β), to induce osteoclast behavior and inhibit osteoblast differentiation. On the contrary, M2 phenotype can secret arginase-1 (ARG-1), interleukin-10 (IL-10) and transforming growth factor-β (TGF-β) to enhance osteoblast migration, proliferation, and differentiation, and extracellular matrix deposition. They can also secrete VEGF and other angiogenesis-related factors to promote vascularization, which is favorable for new bone regeneration and remodeling [51]. Various physical and chemical characteristics of the biomaterials, such as porosity, mechanical properties, surface topography, wettability, geometry, and element composition have osteoimmunomodulation effects [52]. Studies have reported that the modification of the morphological characteristics of biomaterials, especially the designing of the surface with nano-morphological features and porous structures, is one of the most promising strategies to fabricate orthopedic implants with favorable immunoregulatory function. In addition, the analysis of whole genome expression data indicated the involvement of the focal adhesion pathway [49]. which is also one of the most important internal mechanisms of PEGS/20ICPN revealed by proteomic analysis in this study. Additionally, the bioactive elements of materials also play a pivotal role in macrophage polarization and bone formation. Calcium is a critical element involved in the immune response regulation. It was reported that extracellular calcium could modulate macrophage polarization via calcium channels [53]. Furthermore, calcium phosphate (CaP) bioceramics were shown to promote angiogenesis by activating the NFATc1/VEGF pathway of macrophages [54]. Interestingly, in this study, regulation of the immune response was also another significantly enriched pathway involved in PEGS/ICPN-mediated DO. There were also some other potential mechanisms revealed by proteomic analysis such as metabolic process, G protein-coupled receptor activity, and ion binding, which need to be studied further.

**Scheme 2:**
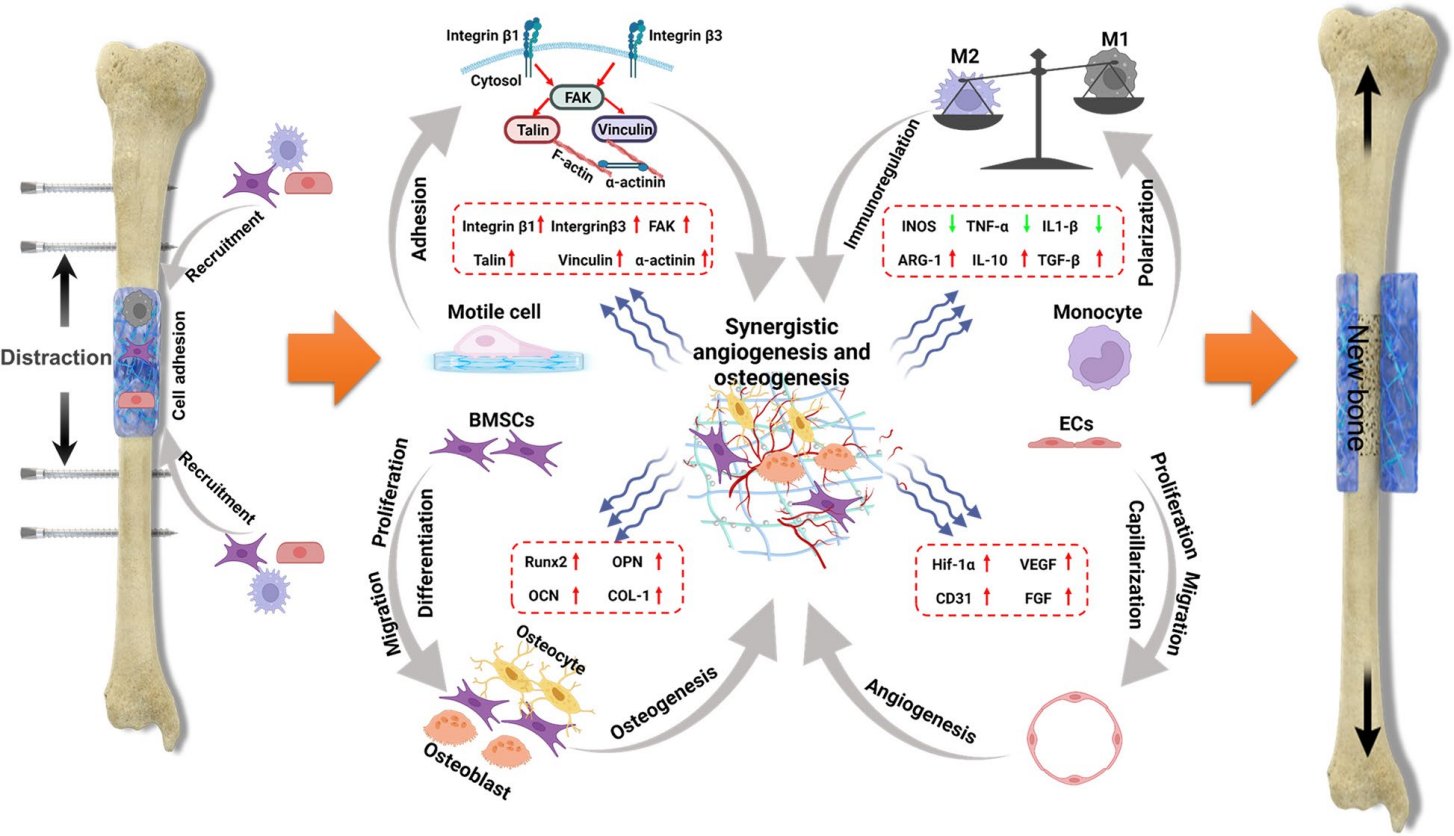
Illustration of the cellular and molecular mechanism of PEGS/ICPN hydrogels for promoting DO process. The introduction of in situ self-assembled CaP nanoparticles to PEGS hydrogel enables spontaneous recruitment of endogenous stem cells and promotes cell adhesion through the FAK pathway. This nano-composite hydrogel also creates a favorable immune microenvironment to promoting bone formation and neovascularization. Eventually, PEGS/ICPN hydrogels promote new bone formation and mineralization during DO through synergistic effects of angiogenesis and osteogenesis

It is well documented that mechanical cues play an essential role in stimulating bone regeneration through a mechanobiology pathway. DO is a dynamic distraction process during which the preserved periosteum transfers mechanical stress to the underling distraction gap, inducing osteogenesis within. However, existing artificial periosteum systems seem unlikely to substitute the function of natural periosteum due to the unique dynamic process of DO. Therefore, we proposed a new design of bionic periosteum to simulate the mechanical transduction of natural periosteum for the application in DO procedure. First, the excellent injectability, robust bone adhesiveness, and superior stretchability of the nanocomposite hydrogel enabled it to transfer mechanical stress to the underlying newly formed bone under distraction force, perfectly meeting the applicable requirements of DO process. Second, the introduction of in situ precipitated CaP nanoparticles mimicked bone-like structure, naturally facilitating endogenous cells recruitment, adhesion, proliferation, migration, and differentiation, subsequently inducing vascularization and new bone formation. Moreover, the porous surface structure and chemical components of PEGS/ICPN hydrogels may exert synergistic effects to materialize the superior pro-osteogenic and pro-angiogenic ability of implants via immunoregulation and cell adhesion (Scheme 2). With this classic DO animal experimental model, the results found here may be valuable in the development of novel periosteum-inspired biomaterials to accommodate dynamic bone regeneration process through a mechanobiology pathway in the future.

## Conclusion

In this study, a periosteum-inspired nanocomposite hydrogel (PEGS/ICPN) was developed to serve as a powerful biomaterial periosteum substitute for accelerating DO procedure. Through the in situ precipitation of CaP nanoparticles in a PEGS polymer network, the prepared homogenous nanocomposite hydrogel exhibits excellent injectability, robust bone adhesion, superior stretchability, and enhanced osteogenic activity, which exquisitely imitates the mechanical transduction of periosteum. More importantly, this bionic periosteum was proven to exert synergistic pro-osteogenic and pro-angiogenic effects both in vitro and in vivo, which relied on the regulation of immune response and cell adhesion. Finally, this bionic periosteum was biological-free and fabricated completely based on biocompatible and biodegradable polymers that are easy to synthesize and handle, suggesting a great translational potential for clinical application (Fig. S14, Supporting Information). Summarily, this periosteum-inspired hydrogel may hold great potential for clinical translation to DO and other medical settings such as fracture healing and periodontal tissue regeneration.

## Supplementary Information

**Additional file 1.** Supporting Information.

## Data Availability

The datasets used and/or analyzed during the current study are available from the corresponding author on reasonable request.
