# Supplementary material for: Periosteum-inspired in situ CaP generated nanocomposite hydrogels with strong bone adhesion and superior stretchability for accelerated distraction osteogenesis
Source: Biomater Res. 2022 Dec 30;26:91. doi: 10.1186/s40824-022-00330-1 (PMC9801553; doi:10.1186/s40824-022-00330-1)
Supplement: Supplementary file 1 — Additional file 1. Supporting Information. [file 40824_2022_330_MOESM1_ESM.docx]

**Supporting Information**

**Title**

**Periosteum-inspired in situ CaP generated nanocomposite hydrogels with strong bone adhesion and superior stretchability for accelerated distraction osteogenesis**

Tengfei Lou^1#^, Kai Chen^2,3#^, Qiyu Luo^1^, Changsheng Liu^2,3^, Yuan Yuan^2,3*^, Cunyi Fan^1*^

^1^Orthopaedic Department, Shanghai Jiao Tong University Affiliated Sixth People’s Hospital, Shanghai 200233, People’s Republic of China

^2^Key Laboratory for Ultrafine Materials of Ministry of Education, and School of Materials Science and Engineering, East China University of Science and Technology, Shanghai 200237, People’s Republic of China

^3^Frontiers Science Center for Materiobiology and Dynamic Chemistry, and Engineering Research Center for Biomedical Materials of Ministry of Education, East China University of Science and Technology, Shanghai 200237, People’s Republic of China

1. **Supplementary Experimental Section**

**1.1 Quantitative proteomics and data analysis**

The distraction callus sample for DIA analysis was grinded by MP9 within 2% SDS buffer containing 50mM DTT for 20min RT and then the supernatants were boiled at 100oC for 5min. The protein sample wsa alkylated for 1 hour at room temperature in the dark by addition of a final concentration of 200mM iodoacetamide (IAA). Then, add five times volume of pre-cooled acetone, and precipitate the proteins overnight in the refrigerator at -20℃. Finally, the protein precipitates were resolved and digested by sequencing grade modified trypsin (Promega) at a protein-to-enzyme of 50:1 at 37 oC overnight. Tryptic peptides were collected by centrifuge for 20 minutes at 14,000g at 20°C. The tryptic peptides were treated with 1% trifluoroacetic acid (TFA), and were purified using the C18 Ziptips, eluted with 0.1% TFA in 50~70% acetonitrile. The eluted peptides were lyophilized using a SpeedVac (ThermoSavant), and resuspended in 1% formic acid 5% acetonitrile. The iRT peptides (Biognosys, Schlieren, Switzerland) were spiked into the sample prior to analysis according to manufacturer instructions. The peptides were re-dissolved in solvent A (A: 0.1% formic acid in water) and analyzed by Orbitrap Exploris 480 with a FAIMS coupled to an EASY-nanoLC 1200 system (Thermo Fisher Scientific, MA, USA）. 2 μL peptide sample was loaded onto a 20 cm analytical column (75 μm inner diameter, 1.7 μm resin (waters BEH)) and separated with 120 min-gradient starting at 6% buffer B (80% ACN with 0.1% FA) followed by a stepwise increase to 20% in 99 min, 32% in 5 min ,80% in 1 min and stayed there for 5 min. The column flow rate was maintained at 250 nL/min with the column temperature of 55°C. The electrospray voltage was set to 2 kV. The mass spectrometer was run under data independent acquisition mode with hybrid data strategy. A survey scan was acquired at 120,000 resolution, normalized AGC target of 3e6 and a maximum injection time of 20 ms. In the DIA MS2 acquisition, variable Isolation window were performed with window widths of 30 m/z (mass range from m/z 350-408 with 2 windows), 10 m/z (mass range from m/z 408-795 with 43 windows), 20 m/z (mass range from m/z 795-985 with 11 windows) and 50 m/z (mass range from m/z 985-1200 with 4 windows). One full scan followed by 20 windows with resolution of 30,000, normalized AGC target of 1e6, and normalized collision energy stepped at 27, 30, and 33. Compensation voltage (CV) of −45 and −65 V were selected and applied to MS/MS scans and the corresponding survey scan. Raw Data of dDIA were processed and analyzed by Spectronaut 14 (Biognosys AG, Switzerland) with default settings. The MS raw data were searched against the rat Uniprot fasta database (29,966 entries, downloaded on May 15, 2018) wihtin the default parameters. In brief, the digestion enzyme was allowed for specfic trypsin enzyme with 2 missed specilized cleavages, and Carbamidomethyl of cysteine specified as a fixed modification and Oxidation of methionine as variable modifications. Retention time prediction type was set to dynamic iRT. Data extraction was determined by Spectronaut based on the extensive mass calibration. Spectronaut will determine the ideal extraction window dynamically depending on iRT calibration and gradient stability. Qvalue (FDR) cutoff on precursor and protein level was applied 1%. Decoy generation was set to mutated which similar to scrambled but will only apply a random number of AA position swamps (min=2, max=length/2). Normalization strategy was set to global normalization. The average top 3 filtered peptides which passed the 1% Qvalue cutoff were used to calculate the major group quantities. After One-way ANOVA Test, different expressed proteins were selected if their p value <0.05 and absolute fold change >1.5. Insights into Gene Ontology (GO) analysis and KEGG enrichment were performed.

1. **Supplementary Figures**


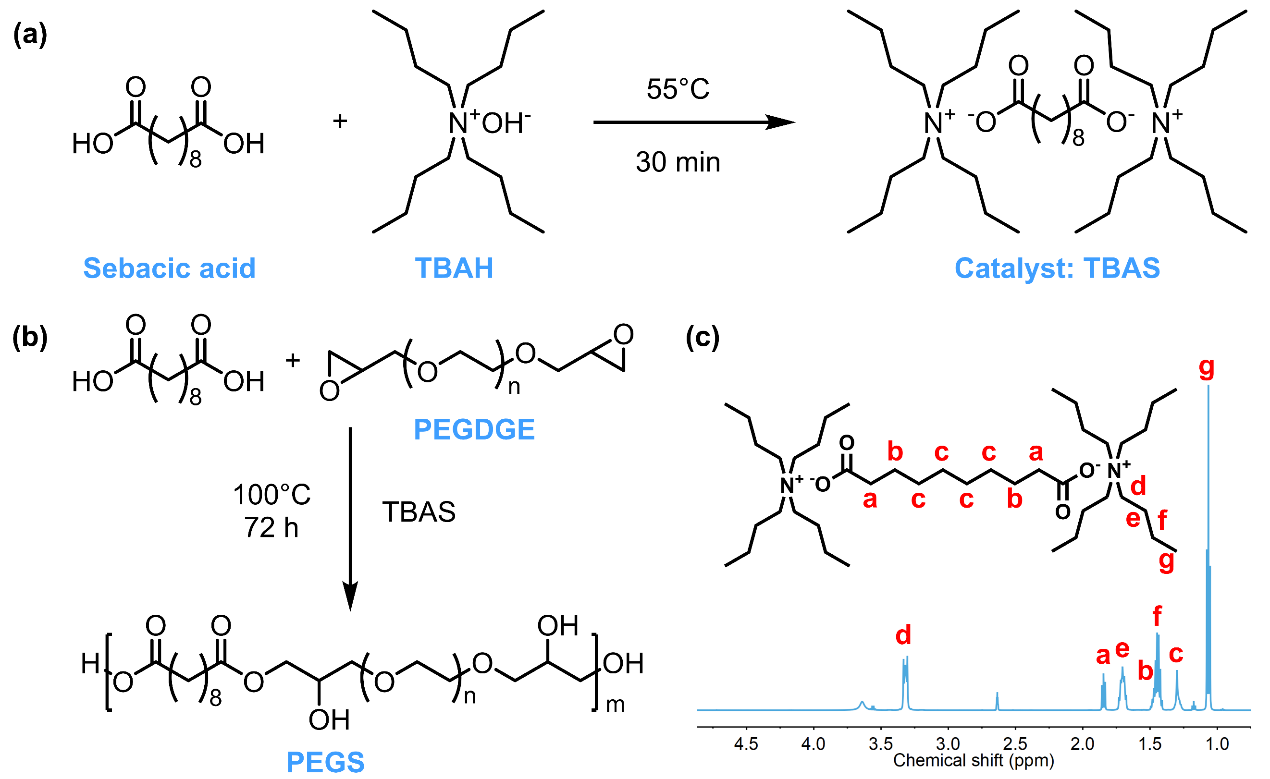


**Figure S1.** a) Synthesis and molecular structure of the catalyst TBAS. b) Synthesis and molecular structure of PEGS. c) ^1^H NMR spectrum of TBAS in DMSO-d6.


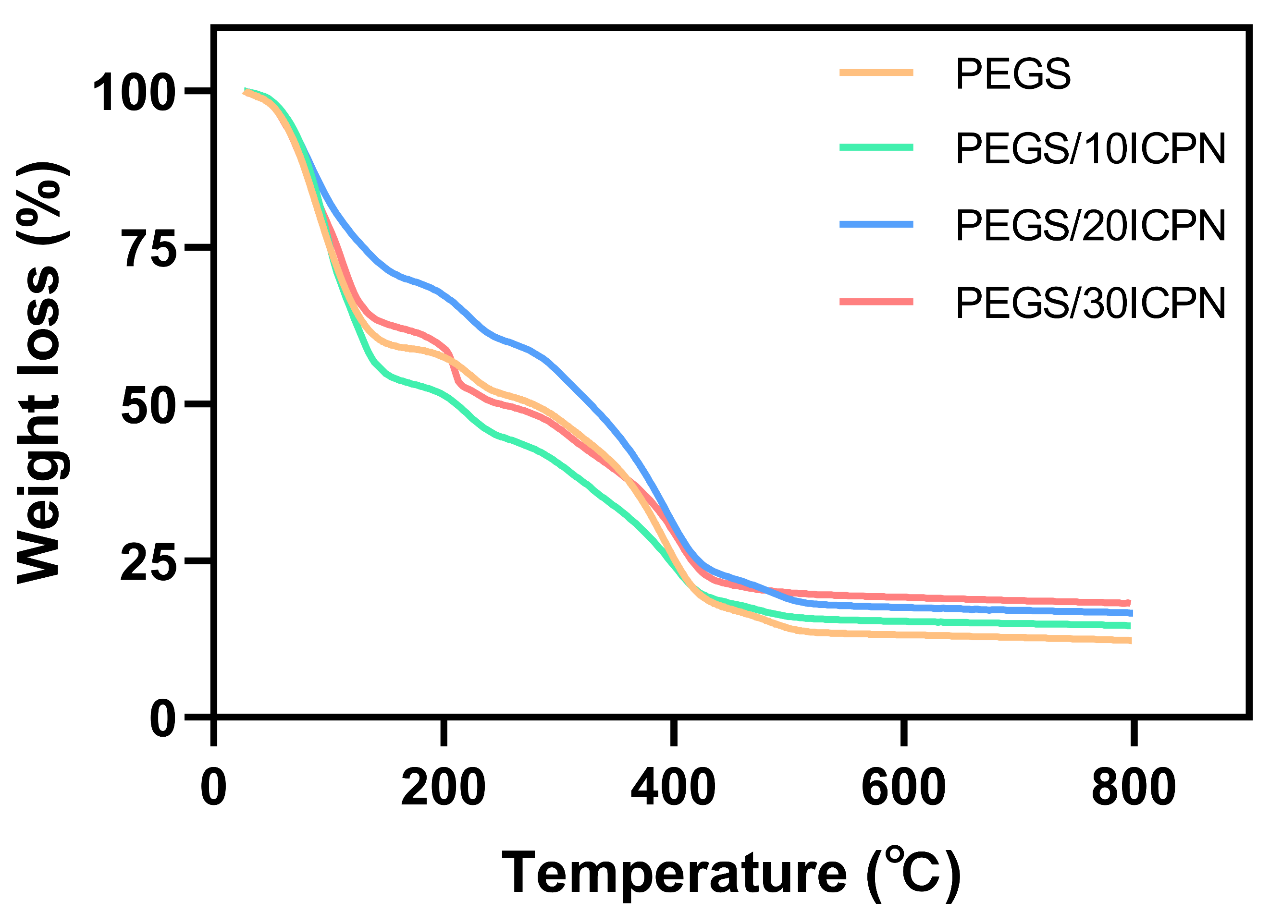


**Figure S2.** TGA results of the nanocomposite adhesive hydrogels in the range of 25-800 ℃.


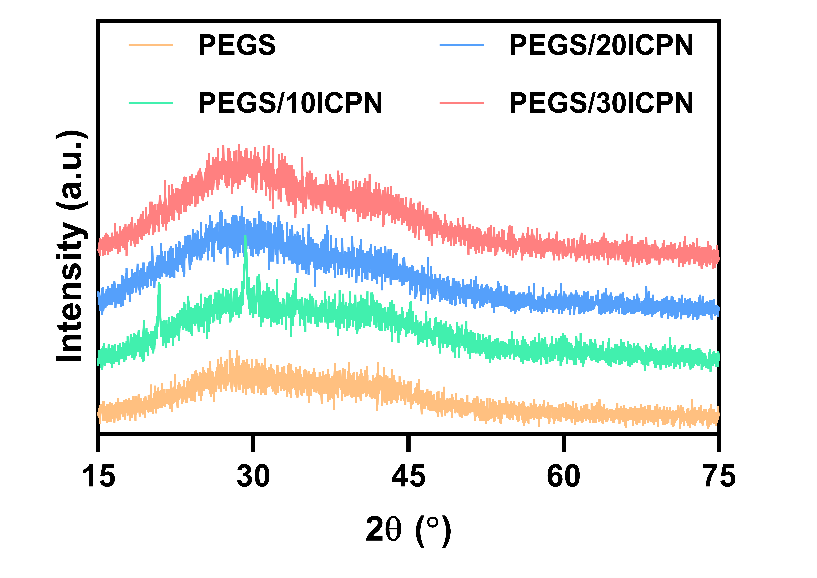


**Figure S3.** XRD patterns of the nanocomposite hydrogels.


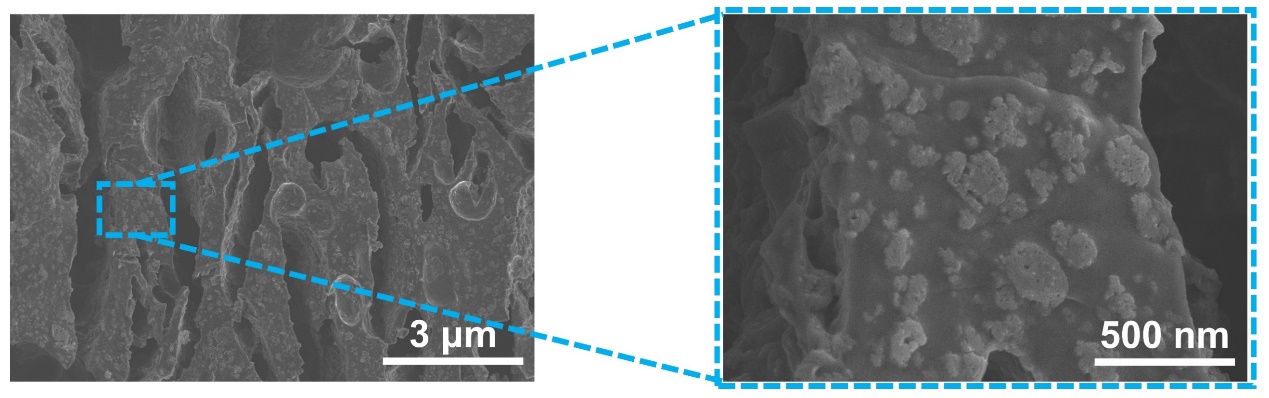


**Figure S4.** SEM images of PEGS/30ICPN hydrogel.


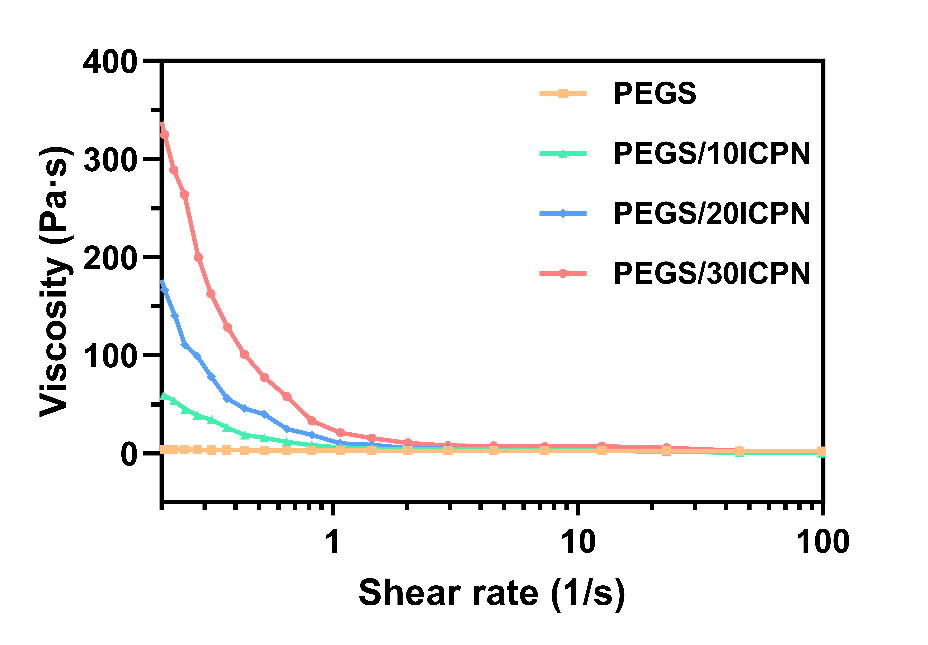


**Figure S5.** Shearing-thinning behavior of the hydrogels in the range of 0.2-100 1/s.


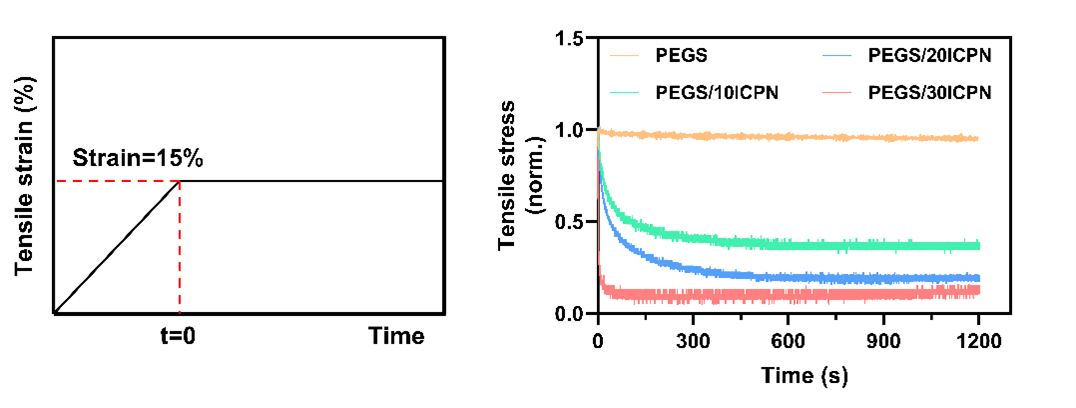


**Figure S6.** Tensile stress relaxation curves at a constant 15% tensile strain of the hydrogels.


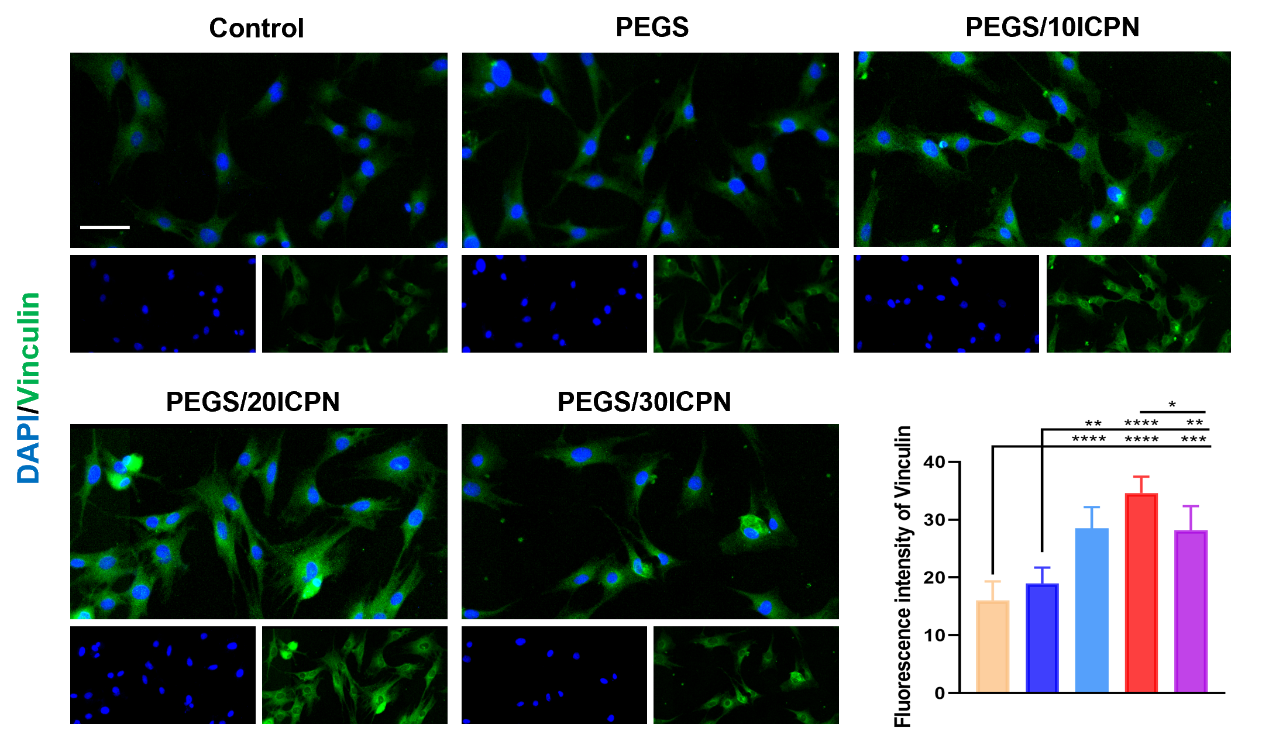


**Figure S7.** Vinculin immunofluorescent staining of BMSCs cultured for 3 days. Vinculin were stained green and the nuclei was stained blue. Scale bar = 25μm.


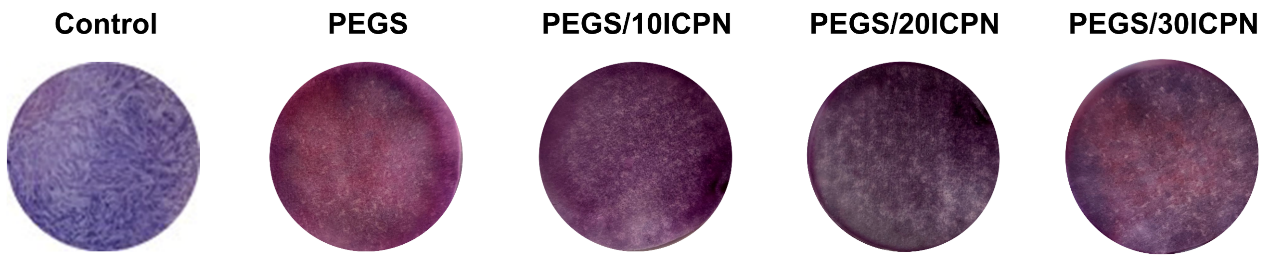


**Figure S8.** ALP staining after 14 days of culture showed that the CaP nanocomposite hydrogels obviously promoted ALP activity, and the most intensive ALP staining could be observed in response to PEGS/20ICPN.


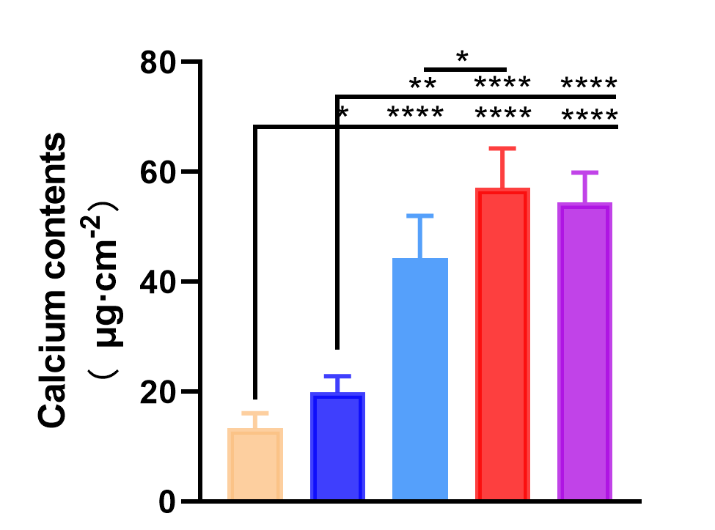


**Figure S9.** Quantitative analysis of calcium concentration in BMSCs at day 14.


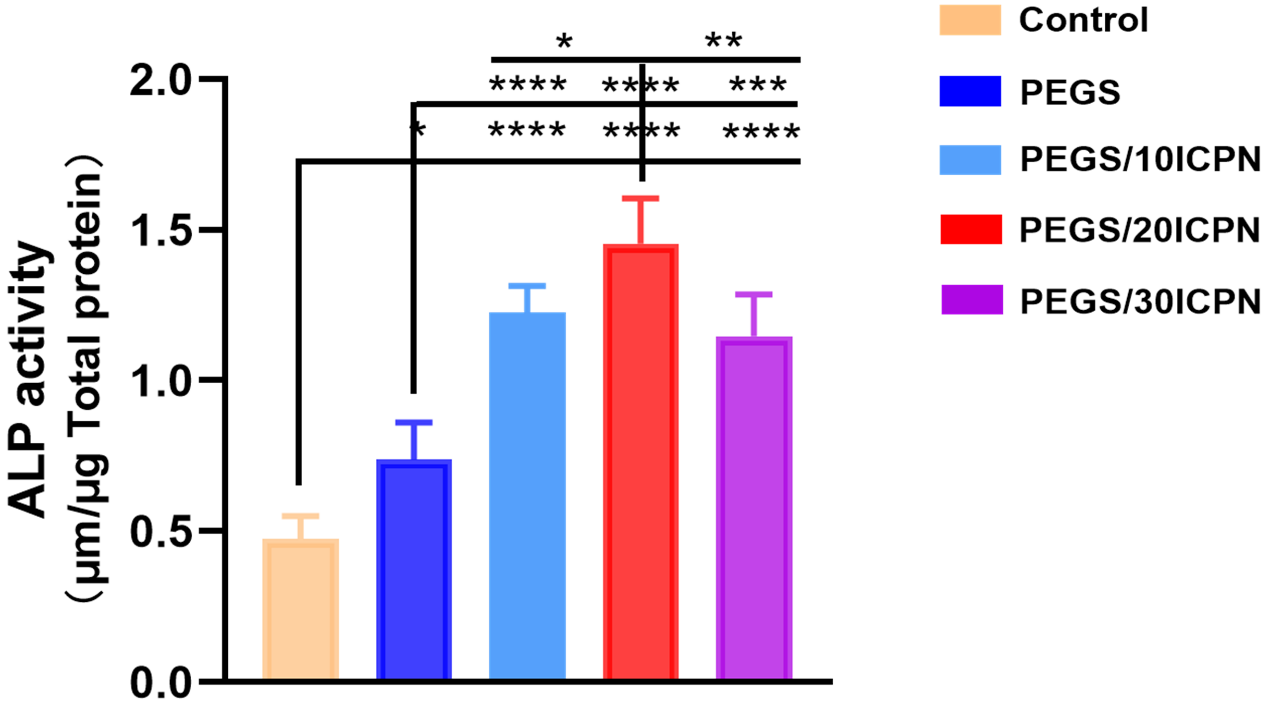


**Figure S10.** ALP activity of BMSCs cultured on different scaffolds for 7 days.


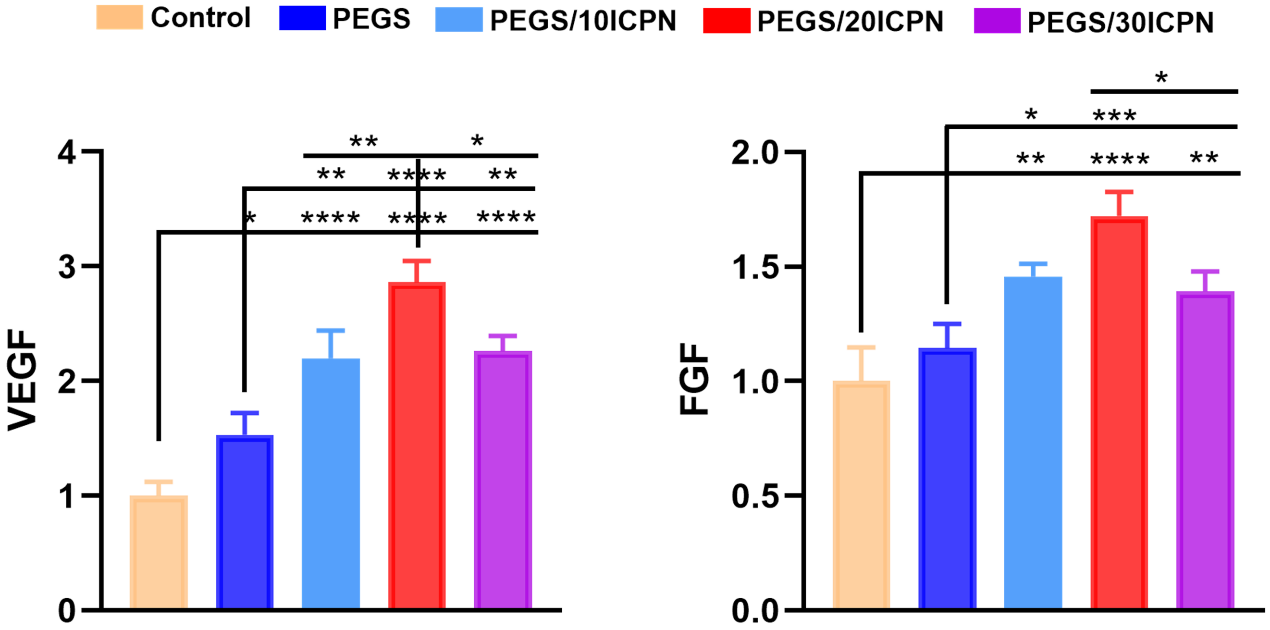


**Figure S11.** Angiogenesis related gene expression (VEGF and FGF).


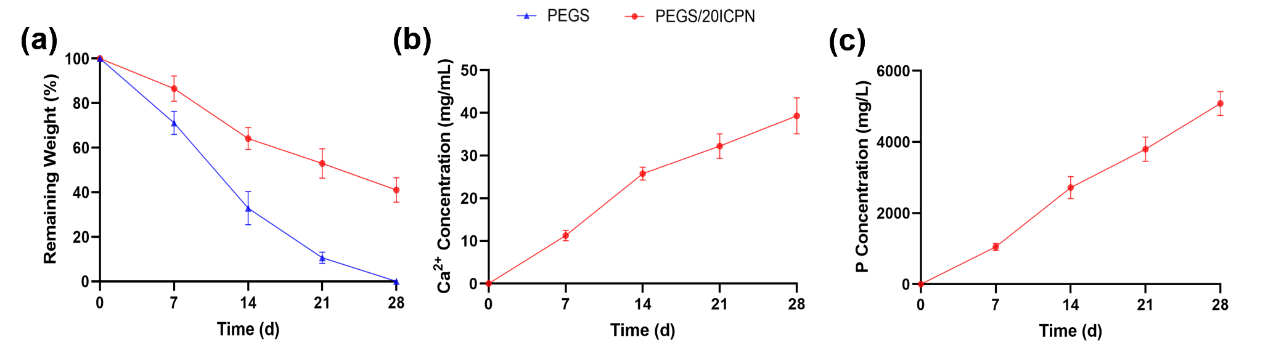


**Figure S12.** Degradation and ion release of the nanocomposite hydrogels. a) Remaining weight during degradation after subcutaneous implantation. b, c) Calcium and phosphorus release curves of PEGS/20ICPN.


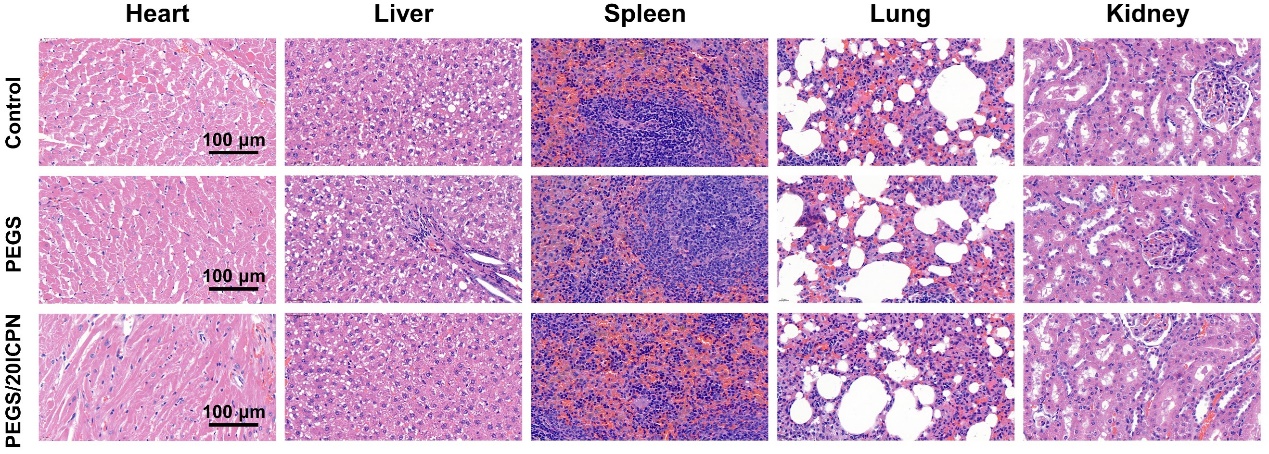


**Figure S13.** Biocompatibility evaluation of PEGS/ICPN Hydrogel in vivo. HE staining of the major organs from rats after treatments (60 days after treatment).


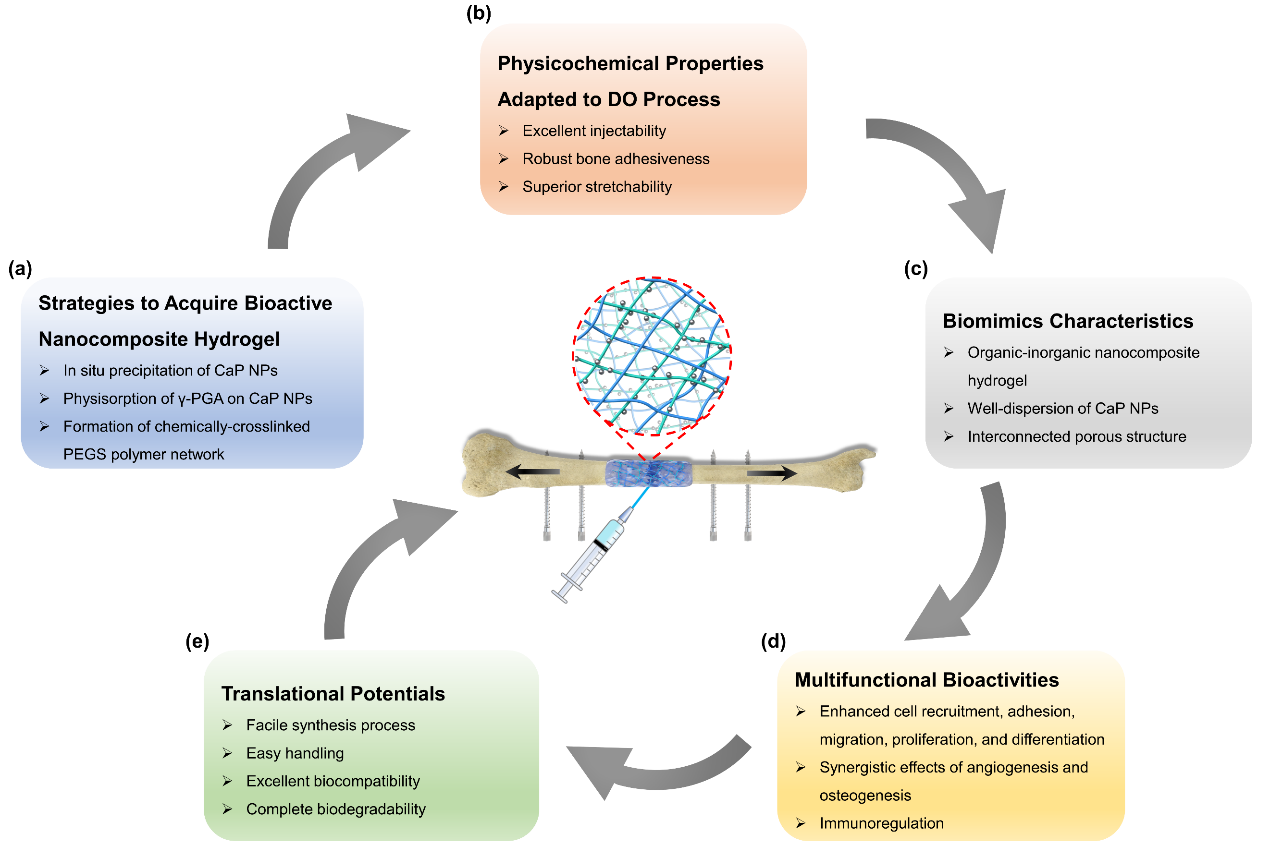


**Figure S14.** Schematic diagram of innovations on the bioactive nanocomposite hydrogel. a) New Strategies to Acquire Bioactive Nanocomposite Hydrogel: The injectable nanocomposite adhesive hydrogel (PEGS/ICPN) is developed through the combination of poly (γ-glutamic acid)-crosslinked PEGylated poly (glycerol sebacate) (PEGS) polymer network and in situ formation of calcium phosphate nanoparticles (ICPNs). b) Physicochemical Properties Adapted to DO Process: The ideal physicochemical properties including excellent injectability, robust bone adhesiveness and superior stretchability enabled the nanocomposite hydrogel to perfectly meet the applicable requirements of DO process. c) Biomimics Characteristics: This nanocomposite hydrogel incorporated with in situ generated CaP nanoparticles allows for a uniform distribution of ICPN NPs in the PEGS matrix. During the degradation process, the CaP bioactive elements can be released continuously. The porous structure of PEGS/ICPN is conducive to the cell ingrowth inward. d) Multifunctional Bioactivities: The introduction of in situ mineralized CaP nanoparticles mimicked bone-like structure, naturally facilitating endogenous cells recruitment, adhesion, migration, proliferation, and differentiation, and subsequently exerting synergistic effects of angiogenesis and osteogenesis via immunoregulation. e) Translational Potentials: From a clinical point of view, the nanocomposite adhesive hydrogel was fabricated completely based on biocompatible and biodegradable polymers with facile synthesis process and easy handling feature, suggesting great translational potentials for clinical application.

| Table S1 Primer sequences of each gene. | | | | |
| --- | --- | --- | --- | --- |
| Target | Forward | | Reverse | |
| RunX2 | TCTTCCCAAAGCCAGAGCG | | TGCCATTCGAGGTGGTCG | |
| Col-1 | CTGCCCAGAAGAATATGTATCACC | | GAAGCAAAGTTTCCTCCAAGACC | |
| OPN | AAGCCTGACCCATCTCAGAA | | GCAACTGGGATGACCTTGAT | |
| OCN | AAACATGGCAAGGTGTGTGA | | AGGTGACCAGGACGTTTTTG | |
| Vinculin | CGCCAGCATTTATTAAGGTCG | | CTGCCACTGTGAGGTATTCCAA | |
| Integrin-β1 | TCAACTGCGATAGGTCCAACG | | CACTGAACACATTCTTTATGCTCTG | |
| Integrin-β3 | CAGATCACTCAAGTCAGCCCTCA | | GGAGAAAGACAGGTCCATCAAGTAG | |
| FAK | GAAAGCAGTAATGAGCCAACCAC | | ACTGAGGCGAAATCCATAGCA | |
| Talin | TGCTCCCAACCTCAAGAGTCAG | | AGTCATGGCTTCACCTAGGACCTTA | |
| TNFα | CCACCACGCTCTTCTGTCTACTG | TGGGCTACGGGCTTGTCACT | |  |
| IL-10 | AGAAGCTGAAGACCCTCTGGATA | TTCATTTTGAGTGTCACGTAGGC | |  |
| TGF-β | CCAACTACTGCTTCAGCTCCACA | GCTTGCGACCCACGTAGTAGA | |  |
| IL-1β | TGTGACTCGTGGGATGATGAC | CCACTTGTTGGCTTATGTTCTGTC | |  |
| HIF-1α | TGATTGCATCTCCATCTCCTACC | GACTCAAAGCGACAGATAACACG | |  |
| VEGF | GGAGGGCAGAATCATCACGA | GCTCATCTCTCCTATGTGCTGG | |  |
| CD31 | ACCAAGATAGCCTCAAAGTCGG | CTGGGAGAGCATTTCACATACG | |  |
| FGF | TGGCACAGTGGATGGGACAA | GTATAAAAGCCCGTCGGTGTCC | |  |
